# Cycloparaffin-rich high density sustainable aviation fuel: pathways from terpenes, terpenoids, and plant oils

**DOI:** 10.1039/d6ra06326h

**Published:** 2026-10-01

**Authors:** Dandu Anitha, Bhushan S. Shrirame, Siddharth Gadkari, Atthasit Tawai, Malinee Sriariyanun, Ao Xia, Vinod Kumar, Sunil K. Maity

**Affiliations:** a Department of Chemical Engineering, Indian Institute of Technology Hyderabad Kandi Sangareddy-502284 Telangana India sunil_maity@che.iith.ac.in +91-40-23016202; b School of Chemistry and Chemical Engineering, University of Surrey Guildford GU2 7XH UK; c The Sirindhorn International Thai-German Graduate School of Engineering, King Mongkut's University of Technology North Bangkok Bangkok 10800 Thailand; d Institute of Engineering Thermophysics, School of Energy and Power Engineering, Chongqing University Chongqing 400044 China; e Department of Engineering Technology, Cullen College of Engineering, University of Houston Sugarland TX 77479 USA Vkumar27@cougarnet.uh.edu +17137432827; f Department of Chemical and Biomolecular Engineering, University of Houston Houston TX 77204 USA

## Abstract

Air transportation is essential for global trade, travel, and military aircraft. Sustainable aviation fuels (SAF) derived from renewable biomass are vital to achieving net-zero carbon-emission goals. Cycloparaffins offer superior aviation fuel properties, such as high density and improved volumetric heat of combustion. Production of cycloparaffin-rich high-density SAF from cineole-based plant oils is a promising approach to produce 100% renewable aviation fuels after blending with synthetic paraffinic kerosene. This review provides an overview of aviation fuels, their key properties, and ASTM-approved SAF technologies with their limitations. The article then focuses on the recent trends in catalytic conversion of terpenes, terpenoids, and plant oils into high-quality SAF. The conversion of terpenoids involves dehydration, hydrodeoxygenation, isomerization, and oligomerization. Conversely, terpenes are processed *via* hydrogenation, dimerization, and cyclopropanation. The review examines the types of catalysts employed in various processes, along with their activity, selectivity, and product yields. It also compares the properties of resulting isomers, dimers, and their mixtures with aviation fuels to assess their suitability as drop-in replacements. Overall, this review presents the manufacturing of high-quality SAF from terpenes and terpenoids as renewable, scalable feedstocks, offering a sustainable solution in reducing greenhouse gas emissions from the aviation sector.

## Abstract 2

The article presents the catalytic conversion of terpenoids, terpenes, and plant oils into cycloparaffin-rich, high-density SAF *via* deoxygenation, hydrogenation, oligomerization, and cyclopropanation over solid-acid and supported metal catalysts.

## Graphical abstract

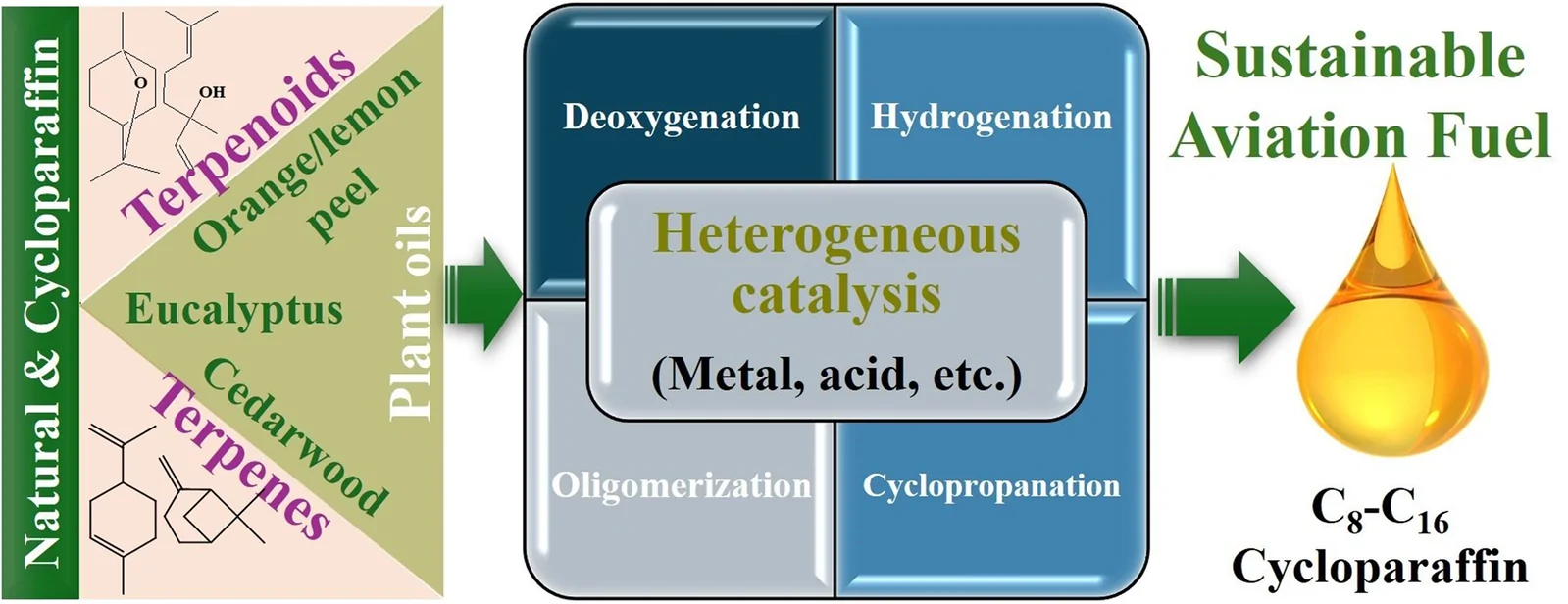

## 1. Introduction

Global energy demand in 2024 increased by 2.2%, surpassing the average growth rate over the past decade, highlighting the need for renewable energy sources.^1^ The rise in energy demands is mainly driven by industrial growth, increased transportation, and urbanization. Notably, the renewables account for the highest growth rate in global energy supply, followed by natural gas, coal, crude oil, and nuclear (Fig. 1A).^1^ However, fossil fuels still dominate the global energy supply despite a notable shift toward cleaner, more sustainable energy options.^1^ Increased energy consumption leads to a 0.8% rise in energy-related CO_2_ emissions, reaching a record 57.1 gigatons in 2023.^2^ The power, transport, and fuel production sectors contributed 14.8, 8.5, and 5.7 gigatons of CO_2_ emissions, respectively (Fig. 1B).^2^ Rising CO_2_ emissions are intensifying global warming and increasing the frequency and severity of extreme weather events, such as wildfires, heatwaves, and flooding. These factors underscore the urgent need to accelerate the transition to sustainable, carbon-neutral renewables to reduce CO_2_ emissions from the energy sector.

**Fig. 1:**
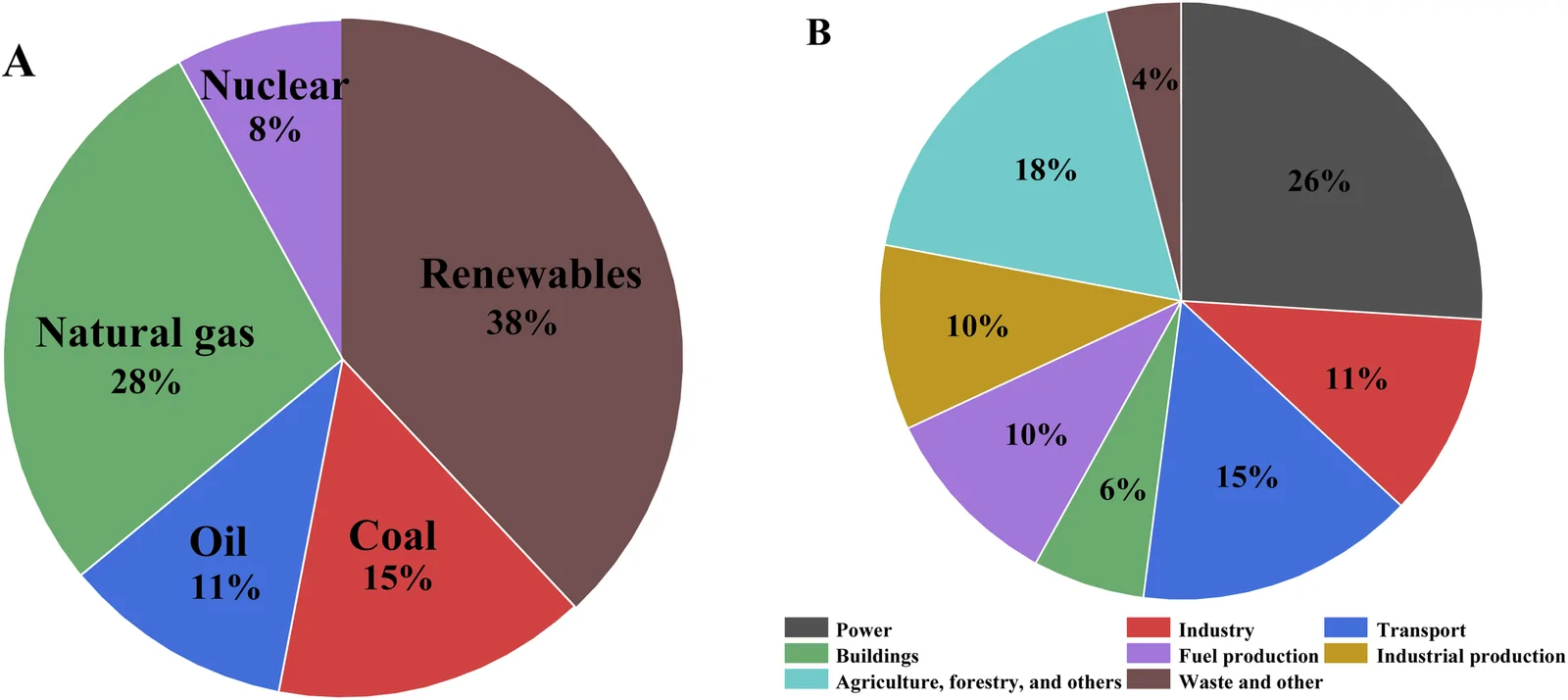
(A) Contribution of energy demand growth in the total energy supply, (B) total CO_2_ emissions from the various sectors.

Among fossil fuels, crude oil demand has surged significantly, mainly driven by the expansion of the aviation and petrochemical industries. In 2024, the petrochemical and aviation sectors accounted for about half of the increase in crude oil demand, with petrochemicals accounting for about 70% of the share.^1^ It is due to the complete dependence of aviation and petrochemicals on fossil fuels. The aviation sector, responsible for 2.5% of global CO_2_ emissions in 2023, has expanded more rapidly than rail, road, and shipping.^3,4^ Notably, the battery-electric and hydrogen fuel technologies offer viable alternatives for road transportation. However, decarbonizing the aviation sector remains challenging due to its reliance on liquid fuels to meet high energy-density, stringent safety norms, and long-haul operational needs. These aspects underscore the pressing need to reduce carbon emissions from the aviation sector by adopting carbon-neutral alternatives. Sustainable aviation fuels (SAFs) derived from renewable biomass are the only solution for reducing the carbon footprint of the aviation sector.^5^ Additionally, SAF offers a unique advantage over other energy solutions, such as hydrogen-based systems and electric propulsion, which require modifications to infrastructures and aircraft engines.^6^

SAFs are composed of C_8_–C_16_ hydrocarbons in the range of aviation fuels and are compatible with existing aircraft engines. The ReFuelEU Aviation Regulation mandates the supply of SAF in Europe, starting at 2% in 2025 and increasing to 70% by 2050.^7^ The International Air Transport Association targets 65% SAF blending in aviation fuels by 2050 as part of its Fly Net Zero initiative.^8^ The ASTM D7566 has approved several SAF production pathways, including Hydro-processed Esters and Fatty Acids (HEFA), Fischer–Tropsch processes (FT), Alcohol-to-Jet (ATJ), catalytic hydrothermolysis synthetic kerosene (CH), hydroprocessing of the fermented sugar, and co-processing pathways.^9,10^ However, these pathways allow only limited blending of SAFs with jet fuels to maintain safety and performance standards.^11^ Notably, the current SAFs are mostly enriched in *n*- and iso-paraffins, which are less dense than aviation fuels, reducing their volumetric net heat of combustion (NHOC). These SAFs also lack high-density cycloparaffins with superior fuel properties. On the other hand, aromatics in SAF contribute to elastomer swelling to prevent fuel leakage.^12^ To address these issues, terpene- and terpenoid-rich plant oils with in-built cycloparaffin structures are considered promising feedstocks for producing cycloparaffin-rich, high-quality SAF.

Terpenes are naturally occurring hydrocarbons found in many plants.^13^ They are composed of isoprene units and are classified as monoterpenes, sesquiterpenes, diterpenes, and triterpenes, depending on the number of carbon atoms. Terpenoids, which contain oxygen, are abundant in essential oils from plants such as eucalyptus, pine, citrus, and chamomile. The SAFs derived from terpenes and terpenoids are comprised of cycloparaffins with higher volumetric energy density, superior NHOC, and better combustion properties, making them strong candidates for next-generation SAF. Furthermore, converting terpenes and terpenoids into cycloparaffin-based SAF enhances fuel density, improves low-temperature properties, and reduces viscosity, addressing key challenges in current SAF options. These plant-based hydrocarbons also promote a circular bioeconomy by reducing dependence on fossil fuels and supporting carbon neutrality.

The previous review articles are focused on the production of SAF from methanol,^14,15^ ethanol,^14^ syngas,^16^ bio-oil,^17^ waste cooking oil,^18^ lignocellulose derivatives,^19^ and CO_2_.^20,21^ Detailed reviews of emerging technologies, performance characteristics, environmental impacts, policies, techno-economic analysis (TEA), and challenges are also available in the literature.^3,22–26^ However, only a limited number of review articles are available on cycloparaffin production from biomass. The cycloparaffin provides advantageous fuel properties, such as high volumetric heat of combustion, high density, and improved operational performance.^12,27^ In a recent review, the production of cycloparaffin-rich SAF from cyclic alcohols, phenolics, cyclic ketones, acyclic ketones, and esters has been discussed in detail.^12^ The use of fast pyrolysis oil and biopolymers, such as lignin, for the production of cycloparaffins has also been comprehensively explored.^28,29^ The C–C coupling of biomass-derived furanics with carbonyl compounds *via* hydroxyalkylation–alkylation and aldol condensation reactions, followed by deoxygenation strategies, has emerged as another approach to produce branched or cycloparaffin-rich SAF.^9,27,30–33^ Catalytic upgradation of α-terpene and related terpenes to value-added products has also been discussed earlier.^34^

However, to the best of our knowledge, the literature is scarce on comprehensive reviews of SAF production from terpene- and terpenoid-rich plant oils. This review thus critically analyses the potential of terpenes, terpenoids, and plant oils for SAF production, focusing on feedstock sources and catalytic conversion methods. The article also includes a brief overview of aviation fuels, their key properties, and ASTM-approved SAF technologies with their limitations. The conversion of terpenoids into SAF involves dehydration followed by dimerization, isomerization, and hydrogenation. Conversely, SAFs are derived from terpenes *via* dimerization, isomerization, hydrogenation, and cyclopropanation. Specifically, this article emphasizes reaction mechanisms, types of catalysts used in different pathways, and their impact on conversion and product distribution. The review also compares the fuel properties of SAF derived from plant oils with those of terpene- and terpenoid-based fuels. Finally, it assesses the techno-economic viability, environmental sustainability, and scalability of these approaches, considering current challenges such as low biological production titters and limited feedstock availability. By leveraging plant-derived hydrocarbons, the aviation sector will advance toward a sustainable, low-carbon future while maintaining energy security and operational efficiency.

## 2. Background

### 2.1. Historical development in aviation fuels

Jet or aviation fuels are used to power aircraft and typically contain C_8_–C_16_ hydrocarbons.^35^ Aviation fuel includes both jet fuel, used in turbine propulsion engines, and aviation gasoline, used in piston engines.^36,37^ Aviation fuel has a long history, starting with the use of low-octane fuel by the Wright brothers in 1903, to the development of modern fuels used in current piston and turbine engines (Fig. 2).^36,38^ During World War I, the aviation fuel had low combustion efficiency. However, the octane number significantly improved between 1920 and 1940, primarily due to military needs.^36,39^ Further evolution of aviation fuel was characterised by the development of Jet Propellant-1 (JP-1) and JP-2, followed by improved blends, JP-3 and JP-4, and naval-grade JP-5.^36,40,41^ These advancements address the safety, performance, and operational requirements. As the commercial aviation sector started booming, kerosene-based Jet A and Jet A-1 became standard aviation fuels.^40^ Further innovation improved aircraft performance by introducing high-energy-density, high-temperature fuels, such as JP-7 and JP-8.

**Fig. 2:**
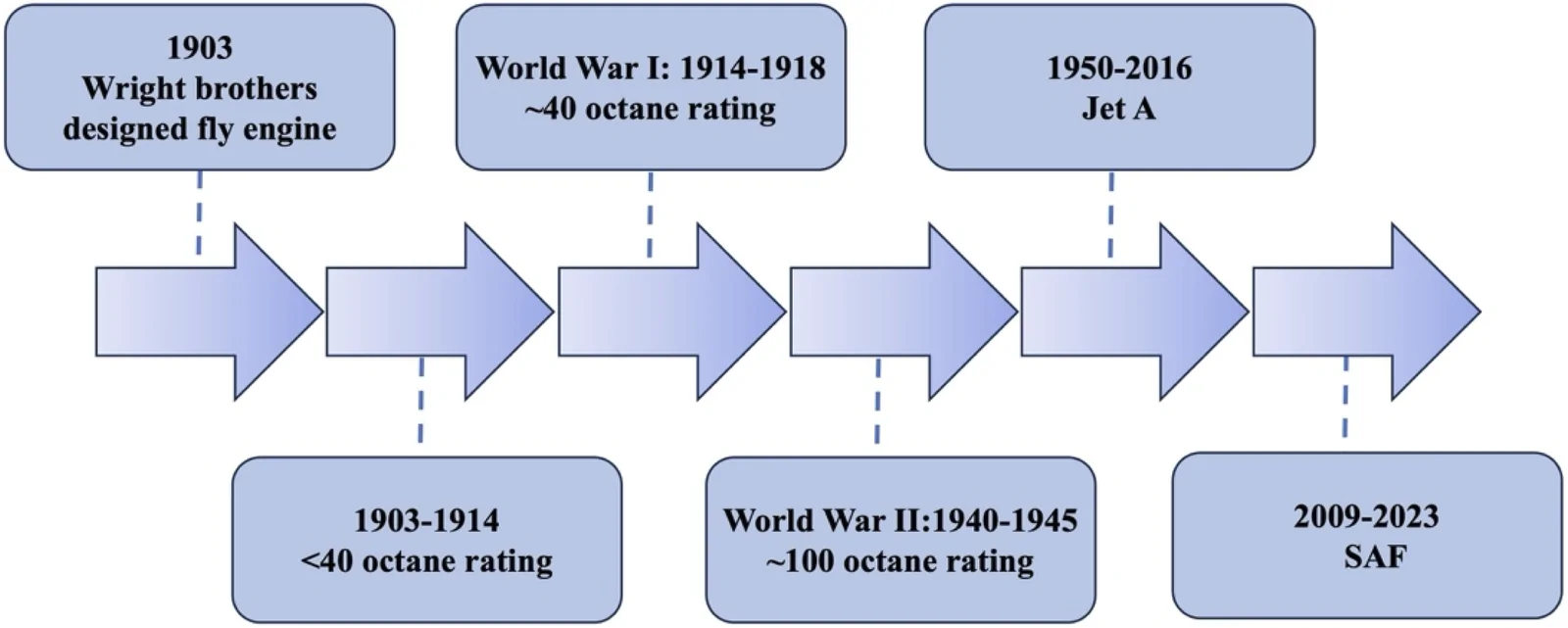
History and evolution of aviation fuel.

### 2.2. Aviation fuel properties and composition

Aviation fuel specifications are more stringent than road transportation fuels due to the unique demands of aircraft engines, high-altitude operations, and strict safety standards. The density and NHOC influence the maximum flight range, as lower values reduce the travel distance per unit volume of fuel. Conversely, a high smoke point is desirable for cleaner combustion, while a higher flash point enhances storage and transportation safety. The excessive aromatics are undesirable due to their strong smoking characteristics.^42^ Since aviation engines operate at altitudes of 11–50 km above sea level and experience extremely low ambient temperatures, freezing point and viscosity are crucial for low-temperature performance. A low freezing point is desirable to prevent fuel blockage, fuel line freezing, and filter clogging.^36,43^ The kinematic viscosity affects nozzle spray characteristics, with lower values enabling faster atomization and shorter ignition delays.^42–44^

Aviation fuel properties depend on the types and molecular weights of hydrocarbons and their composition.^45^ Aviation fuels primarily consist of *n*-paraffins, iso-paraffins, cycloparaffins, and aromatics, with approximately 84% contribution of *n*-, iso-, and cycloparaffins together (Table 1).^35^*n*-Paraffins provide excellent combustion characteristics and short ignition delay, while iso-paraffins lower freezing points. Cycloparaffins, similar in combustion properties to iso*-*paraffins, are denser, with higher volumetric energy density, and have lower freezing points. In contrast, a limited amount of aromatics is necessary for swelling engine elastomers to prevent fuel leakage and increase fuel density.^43,46,47^

**Table 1:** Comparison of hydrocarbon composition of different SAF produced by ASTM approved pathways and Jet A^24,42,47,139^^a^

Components	Hydrocarbons, wt%						Characteristics
	Jet A	HEFA^b^	FT	SIP	ATJ^c^	CH^d^	
*n*-Paraffins	28.8	12.8	19.7	—	—	44	Shorter ignition delay, high specific energy, excellent combustion properties, good stability, poor low-temperature fluidity, and good derived cetane number
Iso-paraffins	39.7	86.9	80.1	98.5	99.8	6.9	Good low-temperature fluidity, variable derived cetane number, and high specific energy
Cycloparaffins	15.5	0.3	0.1	1.3	0.2	32.9	Combustion properties similar to iso-paraffins, high density, lower freezing point, lower H/C ratio, lower specific energy, and higher volumetric energy
Aromatics	14.8	—	—	—	—	8.8	Highest density, essential for swelling elastomers, high smoking, and releases more of its chemical energy as thermal radiation than other hydrocarbons
Olefins	1.2	0.1	0.1	0.2	—	5.1	Good combustion characteristics, highly reactive, and gum forming

a HEFA: hydro-processed esters and fatty acids, FT: Fischer–Tropsch, SIP: synthetic iso-paraffins, ATJ: alcohol-to-jet, CH: catalytic hydrothermolysis synthetic kerosene.

b HEFA of tallow.

c Fuel sourced from the University of Washington.

d Fuel samples sourced from the Air Force Research Laboratory. Ref.: Jet A,^24^ HEFA,^24,139^ FT,^24^ SIP,^24^ ATJ,^24,139^ and CH.^139^

Jet A and Jet A1 are used in commercial aviation engines due to their low freezing point (−40 °C) (Table 2).^48^ Jet A is the dominating aviation fuel in the U.S. and Canada, while Jet A1 is primarily used in Europe, Asia, and other regions outside North America. Jet B (freezing point of −50 °C) is suitable in Arctic regions. JP-5 and JP-8 are widely used military jet fuels in the U.S. and NATO. JP-5 has a high flashpoint with reduced fire hazard and is mainly used in aircraft carriers, amphibious assault ships, and other naval aviation platforms. JP-8 (freezing point of −47 °C) is suitable in extremely cold regions like Canada and Alaska. TS-1 or T-1 is mainly used in Russia and some countries of the former Soviet Union. It is the standard fuel for civil aviation in these regions and is designed to perform in extremely cold conditions (−50 °C).^48^

**Table 2:** Specifications of current aviation fuels^11,140–143^

Standard	Jet A/Jet A1	JP-8	JP-5	Jet B	TS-1
	ASTM D1655-23a	MIL-DTL-83133E	MIL-T-5624R	CAN/CGSB-3.22-2012	GOST 10227-86
Acidity, max, mg KOH per g	0.1	0.015	0.015	0.1	0.7^a^
Aromatics, max, vol%	25	25	25	25	22
Sulfur, max, wt%	0.3	0.3	0.4	0.3	0.25
Sulfur, mercaptan, max, wt%	0.003	0.002	0.002	0.003	0.005
Freezing point, max, °C	−40^b^	−47	−46	−50	−50
Viscosity at −20 °C, max (mm^2^ s^−1^)	8.0	8.0	8.5	—	8.0
Net heat of combustion, min, MJ kg^−1^	42.8	42.8	42.6	42.8	43.12
Smoke point, min, mm	18	19	19	18	25
Density at 15 °C, kg m^−3^	775–840	775–840	788–845	751–802	774^c^
Flashpoint, min, °C	38	38	60	—	28
Initial boiling point, °C	—	—	—	—	155
10 vol%, °C	205	205	206	—	175
50 vol%, °C	—	—	—	145	225
90 vol%, °C	—	—	—	190	270
Final boiling point, °C	300	300	300	270	—

a mg KOH/100 mL.

b −47 °C for Jet A1.

c Minimum value.

### 2.3. Current SAF technologies

SAF, also known as bio-jet, renewable jet, or green jet fuel, is a drop-in alternative to fossil-based jet fuels. SAF is produced from renewable feedstocks, including biomass and other non-fossil carbon sources that meet sustainability criteria, including reduced life-cycle carbon emissions, limited freshwater use, and prevention of deforestation.^8,49,50^ However, readily available edible feedstocks such as vegetable oil, sugar, and starch compete with the food supply, raising concerns about food security. Furthermore, the lack of supportive policies and regulatory frameworks remains a key challenge for the large-scale production and adoption of SAF. In contrast, non-edible feedstocks, including lignocellulosic biomass, inedible oils, used cooking oils, waste animal fats, and industrial or municipal wastes, are considered more sustainable. However, their variable quality poses challenges in SAF manufacturing.^51^ SAF can also be derived from microalgae, but high costs limit their large-scale adoption.^3^ ASTM-approved production pathways and their standard specifications are illustrated in Fig. 3, Tables 3 and S1.

**Fig. 3:**
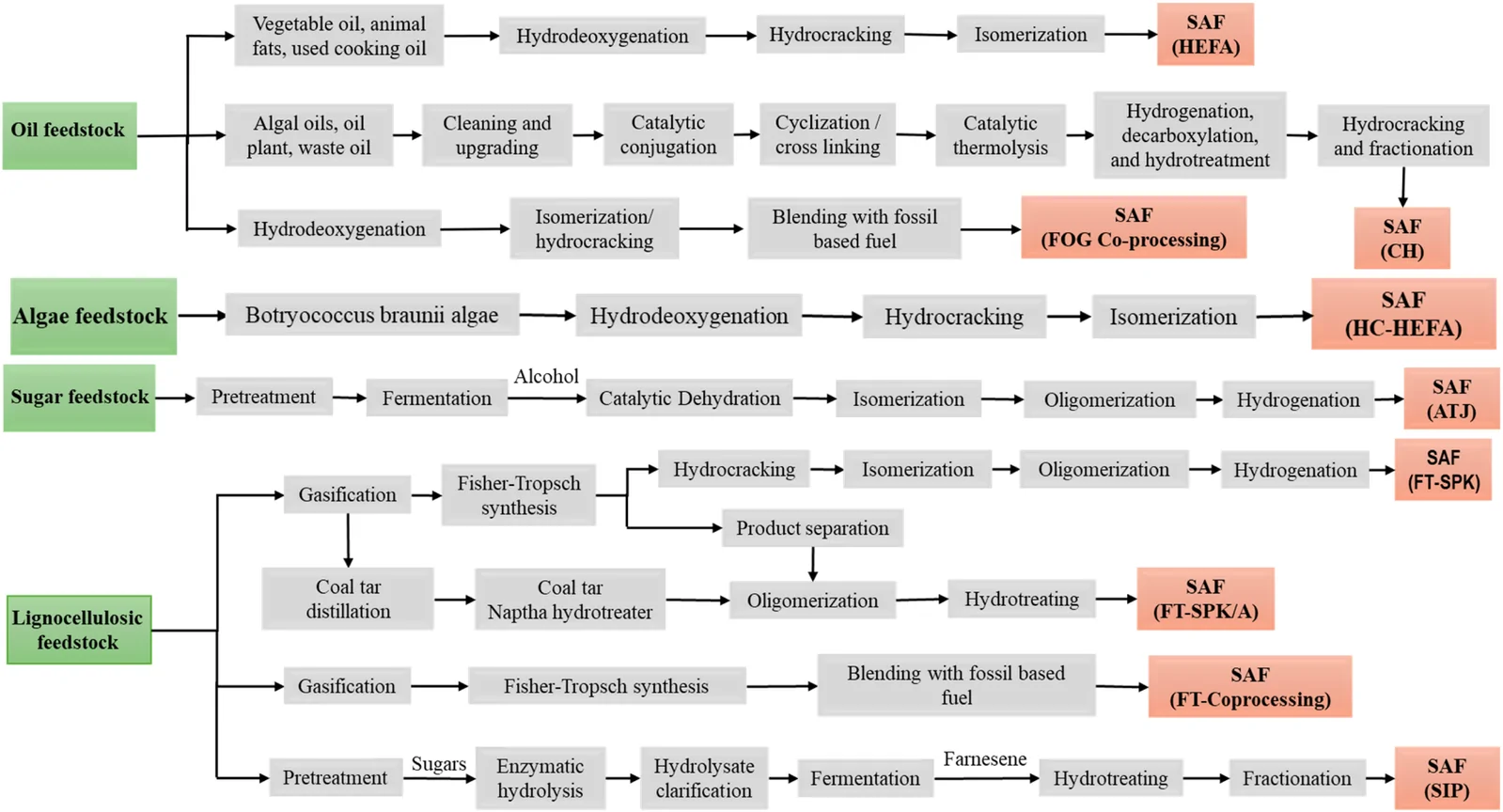
ASTM approved SAF production pathways from biomass.

**Table 3:** Properties of ASTM D7566-approved SAFs^11,36,49,144,145^

Properties	Limit	FT-SPK	HEFA-SPK	HFS-SIP	FT-SPK/A	ATJ-SPK	CH	ATJ-SKA	HC-HEFA-SPK
Molecular formula		C_11_H_23.7_	C_11.3_H_24.4_	C_15_H_32_	—	C_12.5_H_27.1_	C_11.2_H_21.5_	C_11.1_H_23.5_	—
Carbon content, %	—	84.6	84.8	84.9	86.1	85.1	86.1	85	—
*n*-Paraffins, wt%	—	—	—	98	—	—	—	—	—
Olefins, mg Br_2_ per 100 g	Max	—	—	300	—	—	—	—	—
Cycloparaffins, wt%	Max	15	15	—	15	15	—	40	50
Aromatics, wt%	Max	0.5	0.5	0.5	20	0.5	8.4–21.2	8.4–21.2	0.5
Sulfur, mg kg^−1^	Max	15	15	2	15	15	15	15	15
Water, mg kg^−1^	Max	75	75	75	75	75	75	75	75
Nitrogen, mg kg^−1^	Max	2	2	2	2	2	2	2	2
Metals, mg kg^−1^^a^	Max	0.1^b^	0.1^b^	0.1^b^	0.1^b^	0.1^b^	0.1^b^	0.1^b^	0.1^b^
Acidity, mg KOH per g	Max	0.015	0.015	0.015	0.015	0.015	0.015	0.015	0.015
Freezing point, °C	Max	−40	−40	−60	−40	−40	−40	−40	−40
Viscosity at −20 °C, mm^2^ s^−1^	—	—	4.801	14.13	—	4.779	3.977	3.421	—
Net heat of combustion, MJ kg^−1^	Min	—	44.154	43.5	—	43.89	43.202	—	—
Smoke point, mm	—	—	—	—	—	27	22.5	23	25
Density at 15 °C, kg m^−3^	Max	730–770	730–772	765–780	755–800	730–770	775–840	775–840	730–800
Flashpoint, °C	Min	38	38	100	38	38	38	38	38
10% recovered, °C	Max	205	205	250	205	205	205	205	205
50% recovered, °C	—	—	—	—	—	—	—	—	—
90% recovered, °C	—	—	—	—	—	—	—	—	—
Final boiling point, °C	Max	300	300	255	300	300	300	300	300

a Al, Ca, Co, Cr, Cu, Fe, K, Li, Mg, Mn, Mo, Na, Ni, P, Pb, Pd, Pt, Sn, Sr, Ti, V, Zn.

b Per metal.

• Fischer–Tropsch synthetic paraffinic kerosene (FT-SPK). FT-SPK was approved in 2009 with a maximum blending limit of 50 vol%.^52^ This process converts energy crops, lignocellulosic biomass, or solid waste into syngas through gasification, followed by Fischer–Tropsch synthesis to produce fuels.^53^ FT-SPK primarily contains iso-paraffins and *n*-paraffins with low aromatic content and is flexible for feedstock.^3,7,24,47^ However, this process is energy-intensive and involves complex syngas cleaning.^3^

• Hydro-processed esters and fatty acids-synthetic paraffinic kerosene (HEFA-SPK). HEFA-SPK was the second approved process in 2011, with a maximum blending limit of 50 vol%.^23,47,49,50^ It is produced from triglycerides using hydrodeoxygenation (HDO), hydrocracking, and isomerization technologies.^54^ Hydrogen is essential for deoxygenation and saturating double bonds.^55,56^ Hydro-isomerization or hydrocracking is used to enrich iso-paraffins with the desired carbon chain length. HEFA-SPK has a high energy content, a higher cetane number, a lower aromatic content, and the highest energy conversion efficiency among competing routes. However, reliance on oil-based feedstocks limits the versatility and scalability of this process.^57^

• Fischer–Tropsch synthetic paraffinic kerosene with aromatics (FT-SPK/A). FT-SPK/A, a variation of FT-SPK, was approved in 2015 with a maximum blending limit of 50 vol%.^52^ FT-SPK/A is produced by high-temperature Fischer–Tropsch synthesis and enriched with up to 20 wt% alkylated aromatics.^58^ FT-SPK/A is restricted to coal tar feedstock to qualify for ASTM D7566, which narrows its applicability.^3^ The FT-SPK/A are similar to FT-SPK and HEFA-SPK, except for aromatics content and density.^3,59^ The combined processing *via* oligomerization, aromatization, and alkene-aromatic alkylation enables this technology to produce SAF with sufficient aromatics and density suitable to qualify as Jet A1 with 100% synthesized kerosene.^3,24^

• Catalytic-hydrothermolysis synthetic kerosene (CH). CH was approved in 2020 with the highest blending limit of 50 vol%.^52^ It converts vegetable oil, animal fats, greases, and oils into SAF through hydrothermolysis, followed by hydrotreating, hydrocracking, and hydroisomerization.^60^ However, this process operates at elevated temperatures and pressures, with higher energy consumption than HEFA-SPK. The SAF contains *n*-paraffins, iso-paraffins, cycloparaffins, and aromatics.

• Hydro-processed hydrocarbons, esters, and fatty acids synthetic paraffinic kerosene (HC-HEFA-SPK). HC-HEFA-SPK was approved in 2020 with a blending limit of 10 vol%.^52^ This pathway involves hydroprocessing of bio-derived hydrocarbons, unlike free fatty acids and fatty acid esters in HEFA-SPK. However, only lipids from *Botryococcus braunii* algae are recognized as bio-derived hydrocarbons.^47^ This SAF is rich in iso-paraffins.^3,7,24,47^ Although algae with higher growth rates are used, the low blending limit remains a key bottleneck.

• Hydro-processed fermented sugars to synthetic iso-paraffins (HFS-SIP). HFS-SIP was approved in 2014 with a maximum blending limit of 10 vol%.^52^ It uses microbes to convert sugars into hydrocarbons, including long-chain alkenes and β-farnesene.^61^ β-Farnesene is further processed into farnesane, a C_15_ sesquiterpene, through hydrogenation, isomerization, and hydrocracking.^54,58^ Farnesane offers higher energy density than FT-SPK. However, its long carbon chains, high viscosity, and poor combustion performance limit its blending. Additionally, this process is energy-intensive and relies on expensive feedstocks.^23,47,49^

• Alcohol-to-jet synthetic paraffinic kerosene (ATJ-SPK). SAF produced from iso-butanol and ethanol was approved in 2016 and 2018, respectively, with a maximum blending limit of 50 vol%.^53^ This process involves microbial conversion of sugars into C_2_ and C_4_ alcohols, which are then converted into SAF through dehydration, oligomerization, and hydrogenation.^62,63^ However, the high cost of alcohols makes the ATJ-SPK process economically challenging, preventing its large-scale commercial development. Additionally, the ATJ-SPK has a lower energy density, which may affect the aircraft's operating range.^3,23^

• Co-processing. Co-processing of bio-crude with petroleum includes three approved routes: Fischer–Tropsch (FT), Fats, Oils, and Greases (FOG), and Hydroprocessed Esters and Fatty Acids (HEFA). FOG and FT were approved in 2018 and 2020, with blending limits of 5 vol% for FT and 10 vol% for HEFA.^52^ These technologies use HDO and decarboxylation reactions to remove oxygen.^64^ While existing refinery infrastructures are suitable for these processes, challenges remain regarding the types of biogenic feedstocks, blend rates, and product characteristics.

### 2.4. Limitations of the current SAF

Current SAF technologies face significant techno-economic and logistical challenges. Except for CH, FT-SPK/A, and HEFA-SPK, SAFs are rich in acyclic hydrocarbons, resulting in lower density and reduced volumetric heat of combustion than aviation fuels.^12^ These SAFs also cause poor O-ring swellability, which can compromise engine seals, especially in older aircraft.^12^ To overcome these issues, research has focused on developing high-density SAF containing cyclic hydrocarbons. However, aromatics are less suitable due to poor combustion properties, limiting their content to less than 25 vol%. Cycloparaffins are therefore preferred, as adding more than 30 vol% to SPK achieves O-ring swelling like conventional aviation fuel.^12,36^ SAF-range cycloparaffins can be sourced from renewable feedstocks like bio−waste, sugars, terpenes, and terpenoids, providing a sustainable route to high-density SAF with enhanced performance.

### 2.5. Terpenes, terpenoids, and their sources

Terpenes are isoprene (C_5_H_8_) derivatives found in both cyclic and acyclic forms (Fig. 4). They are traditionally used in fragrances, flavours, and medicines due to their distinct aromas and therapeutic properties.^65^ Cyclic terpenes have higher density and NHOC, making them a suitable feedstock for SAF.^13^ Acyclic terpenes can also be converted to high-density SAF *via* ring closure metathesis.^13,66^ Terpenes are classified by the number of isoprene (C_5_) units, with monoterpenes (C_10_), sesquiterpenes (C_15_), diterpenes (C_20_), sesterterpenes (C_25_), triterpenes (C_30_), and tetraterpenes (C_40_) being the most common.^67^ Terpenoids are terpenes with additional functional groups such as carbonyl, hydroxyl, or epoxy (Fig. 4).^13^ They occur naturally and can also be synthesized chemically or biologically *via* metabolic engineering. Their biosynthesis follows two main pathways, depending on the type of organism: bacteria, archaea, protists, or eukaryotes. Many terpenoids are produced by oxidizing or acid-catalysing terpene precursors. Terpenoids are classified in the same way as terpenes.^68^ Terpenes and terpenoids are abundant in various plant parts such as leaves, flowers, and fruits. The larger classes of terpenes, such as sterols and squalene, can be found in animals.^65,69^ Many essential oil-producing plants serve as rich sources of terpenes and terpenoids (Table 4). The most common terpene and terpenoid-rich essential oils include pine, lemongrass, lemon peel, chamomile, lavender, peppermint, tea tree oil, and patchouli. Some terpenes are byproducts of paper manufacturing *via* the Kraft process, such as crude sulphate turpentine.^67^ Terpenes, terpenoids, and essential oils can be converted into cycloparaffin-rich high-quality SAF, as discussed below.

**Fig. 4:**
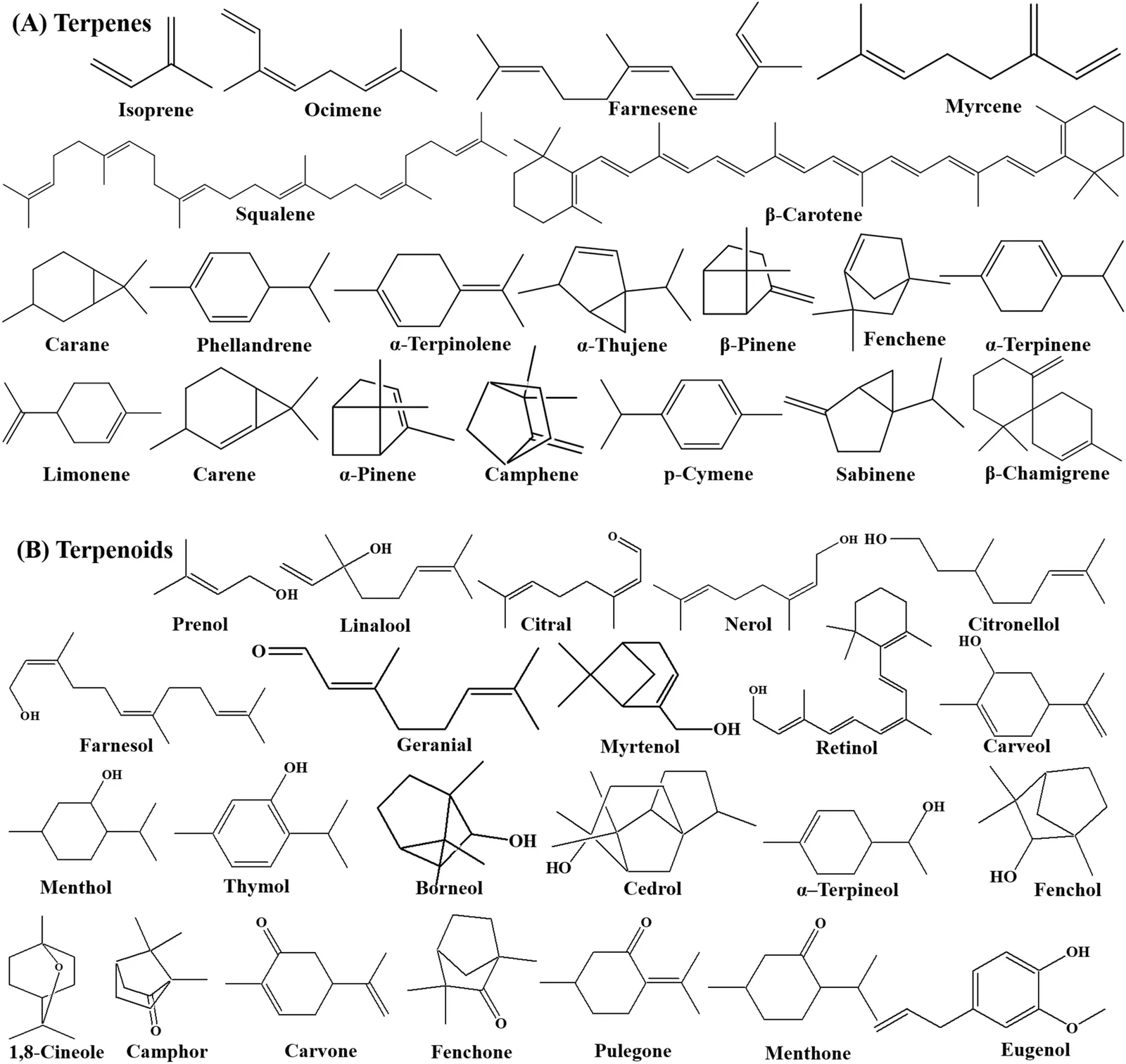
Chemical structure of (A) terpenes and (B) terpenoids.

**Table 4:** Composition of essential oils

Essential oil	Terpene and terpenoids	Ref.
Eucalyptus oil	Cineole (56.5%), α-pinene (16.9%), and d-limonene (5.54%)	146
Chamomile oil	Ocimene, geraniol, citronellol, menthol, and borananol	147
Camphor oil	d-Camphor (46.06%), limonene (9.74%), α-pinene (9.71%), β-myrcene (4.91%), and camphene (4.37%)	148
Peppermint oil	Menthol (40.7%), menthone (23.4%), 1,8-cineole (5.3%), menthyl acetate (4.2%), neomenthol (3.2%), and limonene (2.6%)	149
Tea tree oil	Terpinene-4-ol (40.1%), γ-terpinene (23.0%), α-terpinene (10.4%), 1,8-cineole (5.1%), terpinolene (3.1%), *p*-cymene (2.9%), α-pinene (2.6%), α-terpineol (2.4%), aromadendrene (1.5%), limonene (1.0%), and sabinene (0.2%)	150
Pine oil	β-Pinene (21.5–55.3%), methyl chavicol (8.5–41.5%), α-pinene (3.6–9.6%), δ-3-carene (3.6–6.2%), and α-terpineol (1.4–5.3%)	151
Cedar leaves oil	Terpinene-4-ol (36%), sabinene (19.2%), α-terpinene (5.5%), myrcene (5.5%), α-pinene (1.9%), and α-thujene (2.0%)	152
Cedarwood oil	Thujopsene (47.1%), α-cedrol (10.7%), and widdrol (8.5%)	152
Orange/lime peel oil	Limonene (42.35%), γ-terpinene (15.44%), β-pinene (12.57%), α-pinene (3.12%), neryl acetate (2.2%), and sabinene (2.12%)	121 and 153

## 3. Pure terpenoids to SAF

### 3.1. Eucalyptol (1,8-cineole)

Eucalyptol, a monoterpenoid, also known as 1,8-cineole or 1,8-epoxy-*p*-menthane, is a bicyclic ether. It is a key component of essential oils in many aromatic plants, including eucalyptus oil, and is extracted from plant leaves or stems by steam distillation.^70^ However, its isomer, 1,4-cineole, is present in much lower amounts. Eucalyptol can also be produced from biomass by fungi such as *Hypoxylon* sp. or synthesized chemically.^70,71^ China is the largest producer of eucalyptus oil globally, generating about 17 000 tons of crude essential oil annually, mainly from *Eucalyptus globulus*. Eucalyptol is widely used as a flavouring agent in food, a pharmaceutical for respiratory relief, and a skin stimulant in aromatherapy.^72^ 1,8-Cineole can also be converted into SAF-range C_10_ cycloparaffins, such as *p*-menthane and *p*-cymene, in a batch/flow reactor through C–O ring opening, dehydration, and hydrogenation reactions.^73,74^ Reaction studies in a continuous, fixed-bed reactor using heterogeneous catalysts are essential for commercial process development. In contrast, batch reactor studies are conducted at the laboratory scale to evaluate the performance of various mineral and solid-acid catalysts. The fixed-bed reactor studies were mostly conducted in the vapor phase at relatively high temperatures, whereas batch studies were limited to the liquid phase at lower temperatures.

#### 3.1.1 Flow reactor

The vapor phase catalytic conversion of 1,8-cineole in the absence of hydrogen was conducted in a fixed-bed reactor using Mo, Fe, Co, Cr, and Pd-modified γ-Al_2_O_3_ under both oxygen-free and oxygen environments at 250–500 °C and atmospheric pressure (Table 5).^73^ Products were distributed across three phases: hydrophobic, hydrophilic, and gas. The hydrophobic products comprised aromatic and non-aromatic C_10_ hydrocarbons, with *p*-cymene and dipentene being dominant components. Water was the main hydrophilic product, while H_2_, CO, and CO_2_ were observed in the gas phase. The mechanism follows dehydration of the weak C–O bond to form unsaturated isomers, which then undergo dehydrogenation and double-bond rearrangement to *p*-cymene (Scheme 1).^73^ Undoped γ-Al_2_O_3_, Cr-γ-Al_2_O_3_, and Mo-γ-Al_2_O_3_ were active for both dehydration and dehydrogenation, selectively producing *p*-cymene. However, the reaction was limited to only dehydration over Co-γ-Al_2_O_3_ and Fe-γ-Al_2_O_3_, yielding dipentene as the major product. However, Pd/γ-Al_2_O_3_ enabled both dehydration and dehydrogenation to form *p*-cymene.

**Table 5:** Conversion of 1,8-cineole to SAF-range cycloparaffins in a fixed-bed reactor

Catalyst	Reaction conditions	Major product	Yield (%)	Other (%)	Ref.
γ-Al_2_O_3_	0.5 mL min^−1^ 1,8-cineole, 3 g catalyst, 150 mL min^−1^ carrier gas (7.3% O_2_ in Ar + 5.1% He as an internal standard), 400 °C furnace temperature, 283 °C bed temperature	*p*-Cymene	58	1,8-Cineole (34)	73
Mo/γ-Al_2_O_3_	0.5 mL min^−1^ 1,8-cineole, 3 g catalyst, 150 mL min^−1^ carrier gas (7.3% O_2_ in Ar + 5.1% He as an internal standard), 400 °C furnace temperature, 318 °C bed temperature	*p*-Cymene	52	1,8-Cineole (18)	
Cr/γ-Al_2_O_3_	0.5 mL min^−1^ 1,8-cineole, 3 g catalyst, 150 mL min^−1^ carrier gas (7.3% O_2_ in Ar + 5.1% He as an internal standard), 400 °C furnace temperature, 307 °C bed temperature	*p*-Cymene	25	1,8-Cineole (71)	
Pd/γ-Al_2_O_3_	0.5 mL min^−1^ 1,8-cineole, 3 g catalyst, 150 mL min^−1^ carrier gas (7.3% O_2_ in Ar + 5.1% He as an internal standard), 400 °C furnace temperature, 328 °C bed temperature	*p*-Cymene	94	1,8-Cineole (3)	
Fe/γ-Al_2_O_3_	0.5 mL min^−1^ 1,8-cineole, 3 g catalyst, 150 mL min^−1^ carrier gas (7.3% O_2_ in Ar + 5.1% He as an internal standard), 400 °C furnace temperature, 321 °C bed temperature	Dipentene	45	1,8-Cineole (46)	
Co/γ-Al_2_O_3_	0.5 mL min^−1^ 1,8-cineole, 3 g catalyst, 150 mL min^−1^ carrier gas (7.3% O_2_ in Ar + 5.1% He as an internal standard), 400 °C furnace temperature, 323 °C bed temperature	Dipentene	26	1,8-Cineole (71)	
γ-Al_2_O_3_	0.5 mL min^−1^ flow of liquid cineole, 3 g catalyst, 150 mL min^−1^ Ar, 285 °C	*p*-Cymene	47	Dipentene, *p*-menthadienes, α-terpinene, γ-terpinene, and iso-terpinolene	75
Pd/γ-Al_2_O_3_	0.5 mL min^−1^ flow of liquid cineole, 3 g catalyst, 150 mL min^−1^ Ar, 200 °C	*p*-Cymene	99		
RuNPs@SILP^a^	0.3 mL min^−1^ eucalyptol, 0.535 g catalyst, 80 bar H_2_, 130 °C	*p*-Menthane	>99	*p*-Menthadiene and *p*-menthene	76

a RuNPs@SILP ∼ Ru nanoparticles immobilized on sulfonic acid-functionalized supported ionic liquid phases.

**Scheme 1:**
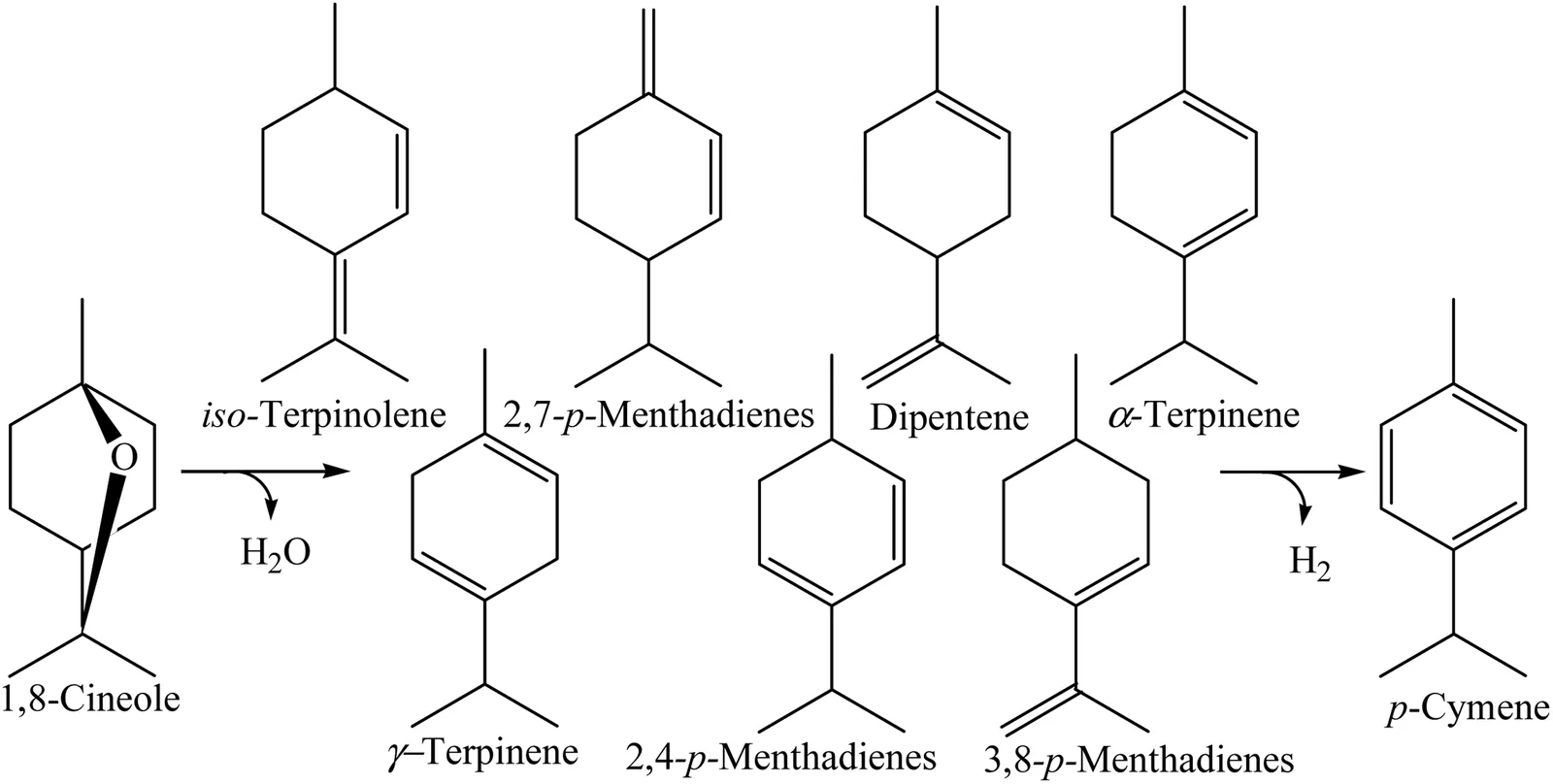
Catalytic conversion of 1,8-cineole to C_10_ cycloparaffins.

Recently, 1,8-cineole was extracted from eucalyptus waste by steam distillation and converted to *p*-cymene over γ-Al_2_O_3_ and Pd/γ-Al_2_O_3_.^75^ The maximum yield of *p*-cymene was 47% over γ-Al_2_O_3_, without hydrogen formation till 400 °C, which might be due to its consumption in the gasification of carbonaceous deposits on the catalyst. Pd/γ-Al_2_O_3_ initiated the reaction at 200 °C with the highest yield of *p*-cymene. The catalytic activity at low temperature was probably due to the reduction of PdO by 1,8-cineole or hydrogen resulting from γ-Al_2_O_3_-catalysed 1,8-cineole conversion. The strong interaction between Pd and γ-Al_2_O_3_ could be another reason for the higher yield over the Pd/γ-Al_2_O_3_. At higher temperatures, the deposition of the carbonaceous matter proliferated, degrading the catalytic activity of γ-Al_2_O_3_, whereas Pd-catalysed gasification helped retain overall catalytic activity of Pd/γ-Al_2_O_3_. Combining both acid and metal functionalities thus facilitated the deoxygenation and hydrogenation of 1,8-cineole.^75^

HDO of eucalyptol was performed using bifunctional Ru nanoparticles (RuNPs) immobilized on acid-functionalized supported ionic liquid phases (SILPs).^76^ The mechanism involves acid-catalysed ether cleavage and dehydration of eucalyptol, followed by metal-catalysed hydrogenation of intermediate dienes and alkenes to *p*-menthane (Scheme 2). The dehydration reaction was the rate-limiting step, with the hydrogenation step being significantly faster. The higher acid loading led to the quicker and more selective conversion of eucalyptol to *p*-menthane. The higher flow of eucalyptol in the gas phase reaction reduced conversion and *p*-menthane selectivity. RuNPs@SILPs exhibited minimal nanoparticle aggregation or leaching. However, water accumulation in the catalyst bed decreased activity over time-on-stream.^76^

**Scheme 2:**
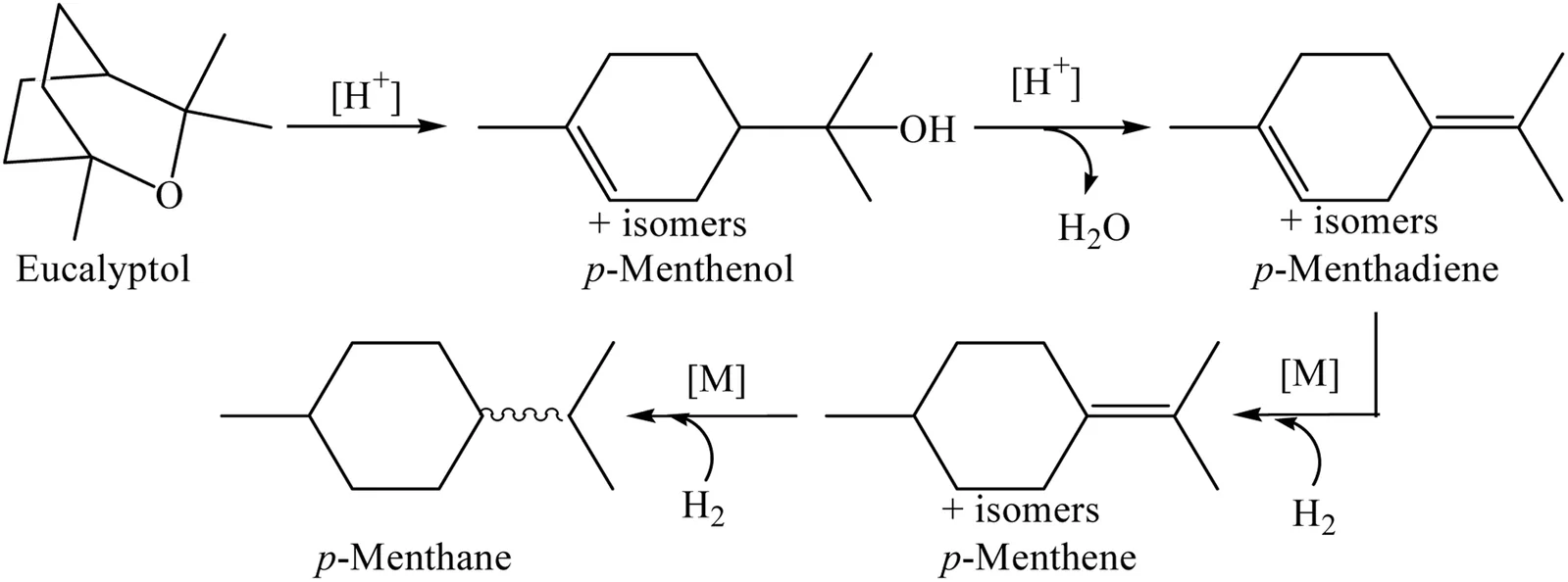
Catalytic conversion of 1,8-cineole to *p*-menthane.

#### 3.1.2 Batch reactor

The catalytic dehydration of 1,8-cineole and 1,4-cineole was investigated over a series of cation-exchange resins, including Amberlyst-15, Nafion SAC-13, and Montmorillonite K10 (Table 6).^77,78^ Catalytic efficacy strongly depends on acid types, acidic strength, and hydrophilicity. Amberlyst-15 showed the highest initial activity, conversion, and dimer yield. Despite the highest acidity, the hydrophilic nature of Nafion SAC-13 favoured water absorption produced by the reaction, requiring high temperatures for its desorption. The initial reaction samples showed a small amount of 1,8-cineole during 1,4-cineole dehydration, while 1,4-cineole was significant during 1,8-cineole conversion. These results imply reversible interconversion between 1,8-cineole and 1,4-cineole (Scheme 3). Although the compounds formed in the 1,8-cineole and 1,4-cineole conversion were largely similar, their distribution differed significantly. The 1,4-cineole conversion mechanism involved ring opening to terpinene-1-ol, followed by dehydration to α-terpinene and γ-terpinene. However, 1,8-cineole contains an oxygen atom linkage to a central carbon of the exocyclic isopropylidene group. Therefore, 1,8-cineole ring opening resulted in the formation of α-terpineol. α-Terpineol, upon dehydration, gave terpinolene and limonene. Montmorillonite K10 additionally showed a significant quantity of another product, *p*-cymene, during the deoxygenation of 1,4-cineole.^77,78^ It was formed by the dehydrogenation of the terpene intermediates. The conversion of 1,8-cineole to *p*-menthane was further studied using homogeneous and heterogeneous catalysts in a biphasic reactor using water-cyclohexane solvent.^74^

**Table 6:** Catalytic conversion of 1,8-cineole to SAF-range cycloparaffins in a batch reactor

Catalyst	Reaction conditions	Conv. (%)	Desired product	Yield (%)	Other (%)	Ref.
Amberlyst-15	5 g 1,8-cineole, 0.25 g catalyst, 85 °C, and 24 h	83	Dimers	47	a	77
Nafion SAC-13		70	Dimers	4	b	
MMTK-10		38	Dimers	7	c	
H_2_SO_4_	0.15 g 1,8-cineole, 25 mL H_2_O, 10 mL cyclohexane, 25 mL 0.5 mol L^−1^ H_2_SO_4_ or H_3_PO_4_, 100 °C, and 2 h	94	Dimers	>40	d	79
H_3_PO_4_		35	Dimers	7	e	
Amberlyst-15 + Pd/C	23.31 g 1,8-cineole, 2.38 g Amberlyst-15, 0.26 g 10% Pd/C, 500 psi H_2_, heated 50 °C for 5 h and 100 °C overnight	—	*p*-Menthane	95.3	*p*-Cymene (4.7)	74
Pd/AC	0.16 g 1,8-cineole, 0.01 g Pd/AC, 5 mL cyclohexane, 120 °C, 1 MPa H_2_, and 1 h	5	*p*-Menthane	∼3	—	
Trifluoroacetic acid	0.16 g 1,8-cineole, 0.5 M trifluoroacetic acid, 25 mL H_2_O, 120 °C, 1 MPa H_2_, and 1 h	>98.5	*p*-Menthane	< 4.5	Terpinolenes or terpinenes	
Trifluoroacetic acid + Pd/AC	0.16 g 1,8-cineole, 0.01 g Pd/AC, 0.5 M trifluoroacetic acid, 25 mL H_2_O, 5 mL cyclohexane, 120 °C, 1 MPa H_2_, and 1 h	98	*p*-Menthane	99.9	—	
Acetic acid + Pd/AC	0.16 g 1,8-cineole, 0.01 g Pd/AC, 25 mL H_2_O containing an acetic acid, 5 mL cyclohexane, 120 °C, 1 MPa H_2_, and 1 h	3.2	*p*-Menthane	3.1	—	
Sulfuric acid + Pd/AC	0.16 g 1,8-cineole, 0.01 g Pd/AC, 0.1M H_2_SO_4_, 25 mL H_2_O, 5 mL cyclohexane, 120 °C, 1 MPa H_2_, and 1 h	40	*p*-Menthane	38	—	
RuNPs@SILP-1.00^f^	7.2 mmol eucalyptol, 0.225 g catalyst, 120 bar H_2_, 125 °C, and 12 h	100	*p*-Menthane	100		76
SILP-1.00	2.4 mmol eucalyptol, 0.075 g catalyst, 120 bar H_2_, 150 °C, and 12 h	>99	*p*-Menthadiene	>99		
RuNPs@SILP-1.00	2.4 mmol eucalyptol, 0.075 g catalyst, 120 bar H_2_, 150 °C, and 12 h	>99	*p*-Menthane	>99		
RuNPs@IL-SO_3_H	2.4 mmol eucalyptol, 0.075 g catalyst, 120 bar H_2_, 150 °C, and 12 h	>99	*p*-Menthane	85	*p*-Menthene (13) and *p*-menthadiene (2)	
La(OTf)_3_ or Yb(OTf)_3_	0.925 g 1,8-cineole, 0.0127 g Pd/C, 0.5 mol% metal triflate, 100 °C, and 0.5 h	0.2	*p*-Menthanes and *p*-cymene	—	Terpinene isomers	80
Zr(OTf)_4_		33.5				
Hf(OTf)_4_		46.0				
Hf(OTf)_4_	0.925 g 1,8-cineole, 0.0127 g Pd/C, 0.5 mol% metal triflate, 1 M *n*-octane solvent, 100 °C, and 0.5 h	49.7				
Amberlyst-15 + Pd/C	23.31 g 1,8-cineole, 2.38 g Amberlyst-15, 0.26 g Pd/C, 500 psi H_2_, 50 °C for 5 h, and 100 °C for overnight	100	*p*-Menthane	95	*p*-Cymene (5)	81

a Terpinolene = 11, α-terpinene = 11, γ-terpinene = 7, and limonene = 4.

b Terpinolene = 21, α-terpinene = 26, γ-terpinene = 15, and *p*-cymene = 1.

c Terpinolene = 13, α-terpinene = 8, γ-terpinene = 4, 1,4-cineole = 3, and limonene = 1.

d α-Terpinene = ∼22, γ-terpinene = ∼8, *p*-cymene = ∼4, *p*-menthane-3,8-diene = ∼3, terpinolene = ∼10, dipentene = ∼1, and other = ∼12.

e α-Terpinene = ∼18, γ-terpinene = ∼4, *p*-cymene = ∼2, *p*-menthane-3,8-diene = ∼1, terpinolene = ∼6, dipentene = ∼1, 1,4-cineole = ∼2, and other = ∼3.

f RuNPs@SILP-1.00 ∼ Ru nanoparticles immobilized on sulfonic acid-functionalized supported ionic liquid phases.

**Scheme 3:**
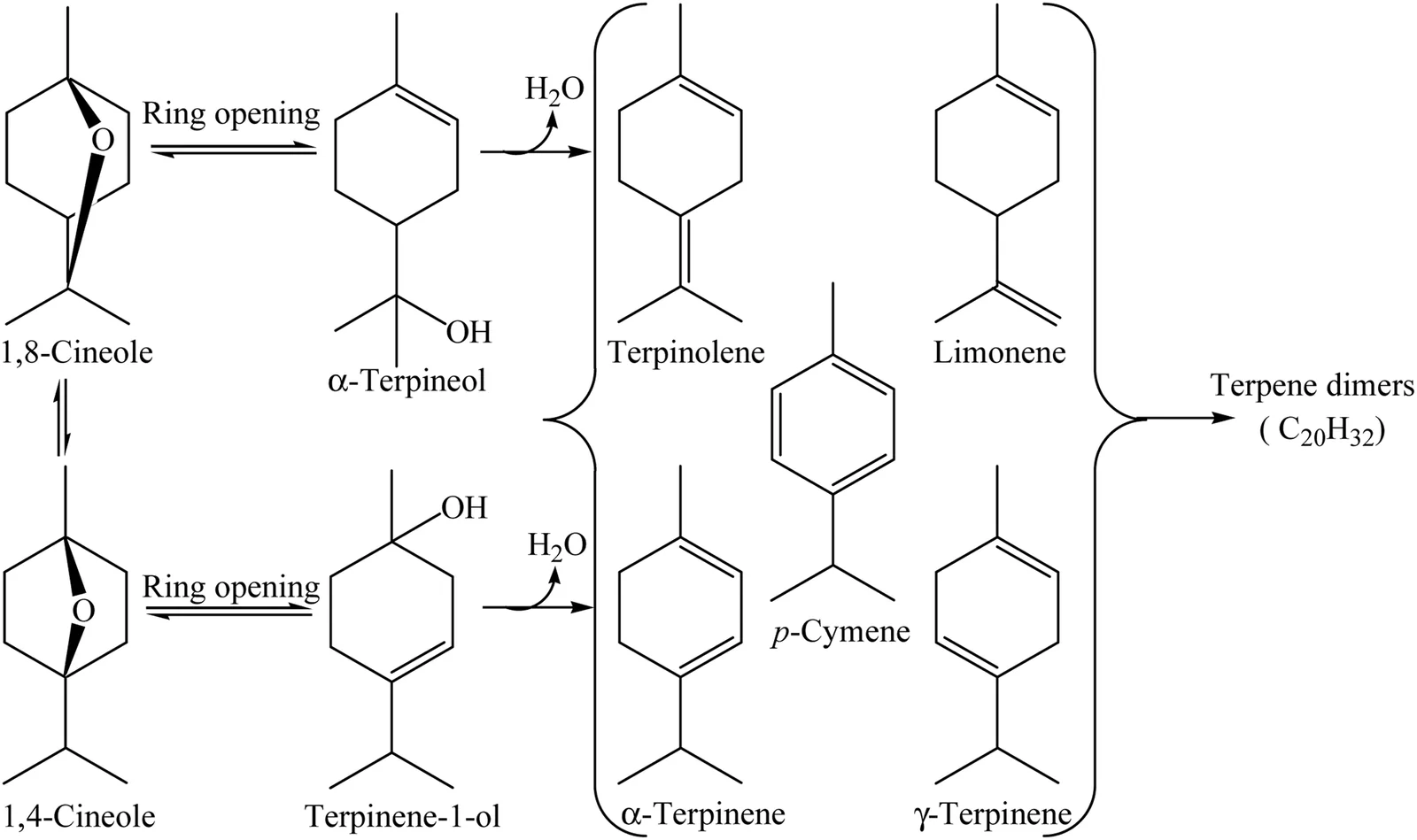
Mechanism of dehydration of 1,8-cineole and 1,4-cineole.

The one-pot conversion of 1,8-cineole was recently studied in a biphasic reactor in the presence of hydrogen using hydrophobic (Pd/AC) and hydrophilic (trifluoroacetic acid, TFA) catalysts in water-cyclohexane solvent.^74^ This approach allowed easy separation of hydrophobic products, minimizing solid residue formation. When the reaction was performed in a monophasic reactor using Pd/AC in cyclohexane and TFA in water, *p*-menthane yield was merely ∼3% and <4.5%, respectively. TFA showed >98.5% 1,8-cineole conversion, but terpinolenes/terpinenes were the major products.^74^ TFA catalysed the ring-opening and dehydration reactions. The low *p*-menthane yield and excessive solid residue formation over TFA were due to the absence of metal sites required for terpinolenes hydrogenation. In contrast, Pd/AC catalysed the hydrogenation of terpinolenes to *p*-menthane. Adding both Pd/AC and TFA to the monophasic reactor thus improved *p*-menthane yield to ∼30% and ∼43% for the aqueous and organic phase reaction, respectively. In contrast, catalytic activity was significantly improved in the biphasic system using Pd/AC and TFA catalysts, with nearly 98% 1,8-cineole conversion and 99.9% *p*-menthane selectivity.^74^ Other Brønsted acid catalysts, such as acetic and sulfuric acids, were also investigated at the same initial pH values, which showed lesser conversion and *p*-menthane yield.

The biphasic reaction of 1,8-cineole over TFA followed second-order kinetics with or without the hydrogenation step.^74^ 1,8-Cineole first underwent acid-catalysed ring-opening, which was subsequently dimerised with another 1,8-cineole molecule. Density functional theory suggested that the dimerization reaction was the rate-limiting step.^74^ According to their mechanism, Brønsted acid (H^+^) first interacts with an oxygen atom, facilitating the C–O bond cleavage. Subsequently, the conjugate base of TFA attacks the carbon of the C–O bond, opening the cyclic ether ring. The hydroxyl group then participates in a nucleophilic substitution reaction with another 1,8-cineole molecule, forming a dimer intermediate (Scheme 4). In the presence of water, the dimer undergoes hydrolysis to produce terpin, which is then dehydrated and hydrogenated to form hydrocarbons.^74^ The product distribution was strongly influenced by hydrogen partial pressures, with *p*-cymene and *p*-menthane being the major products at lower and higher pressures, respectively.^74^

**Scheme 4:**
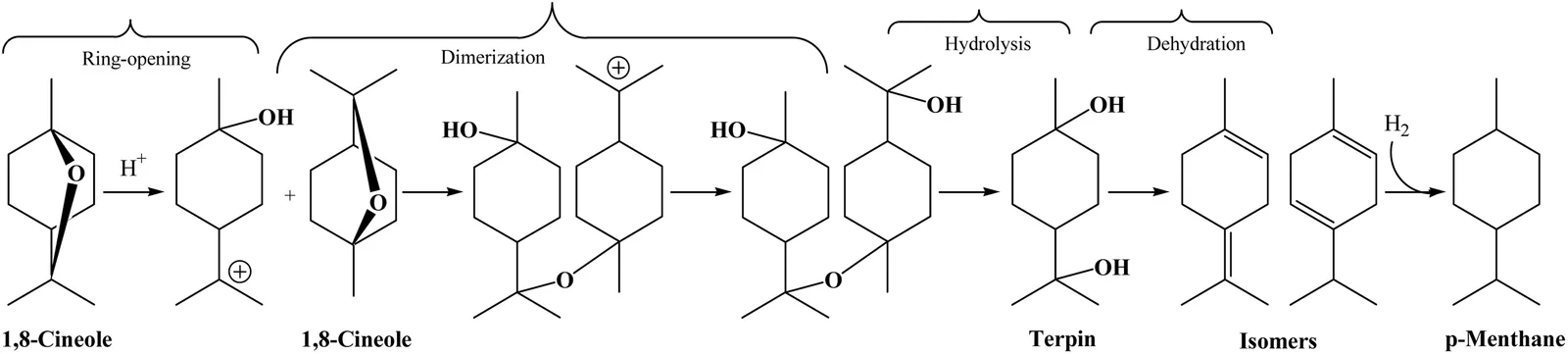
Catalytic conversion of 1,8-cineole to *p*-menthane.

The density of *p*-menthane (808.6 kg m^−3^) is not sufficient for high-performance engines. A biphasic catalytic process was thus tailored for converting 1,8-cineole into high-density terpene dimers.^79^ Homogeneous Brønsted acids of varying acid strengths, such as acetic acid, TFA, H_3_PO_4_, and H_2_SO_4_, were used for the 1,8-cineole conversion. Acetic acid exhibited no catalytic activity due to its low acidity. Surprisingly, no 1,8-cineole conversion was observed over TFA despite its stronger acidity than H_3_PO_4_, possibly due to its evaporation from the reaction mixture. H_3_PO_4_ also showed poor catalytic performance in both aqueous and biphasic reactions. However, 1,8-cineole conversion over H_2_SO_4_ was nearly 50% for the aqueous phase reaction, yielding only monomer products. Although the organic-phase reaction improved 1,8-cineole conversion to 100%, only small amounts of dimers were formed. On the contrary, the biphasic reaction using water-cyclohexane solvent slightly reduced 1,8-cineole conversion to 94%, while dimer carbon yield was more than 40%. Lower water volume and higher H_2_SO_4_ concentration in water were reported to promote dimerization reactions.^79^ The 1,8-cineole conversion to terpene dimers involves a sequence of ring opening and dehydration reactions, generating terpinene isomers, which subsequently undergo dimerization (Scheme 5). 1,8-Cineole protonation first generates reactive intermediates. These intermediates undergo deprotonation *via* α-H elimination to form C=C bonds, forming two distinct terpineols. γ-Terpineol forms from both 1,8-cineole or 1,4-cineole, allowing their interconversion. Terpineols are unstable in acidic environments and tend to dehydrate, creating positively charged olefins, which further deprotonate to produce dienes. The organic phase promotes dehydration, favouring diene formation. The ring-opening of 1,4-cineole is faster than that of 1,8-cineole, resulting in α-terpinene as the primary intermediate. The α-terpinene and other dienes subsequently underwent cross-coupling, yielding a mixture of terpene dimers.^79^

**Scheme 5:**
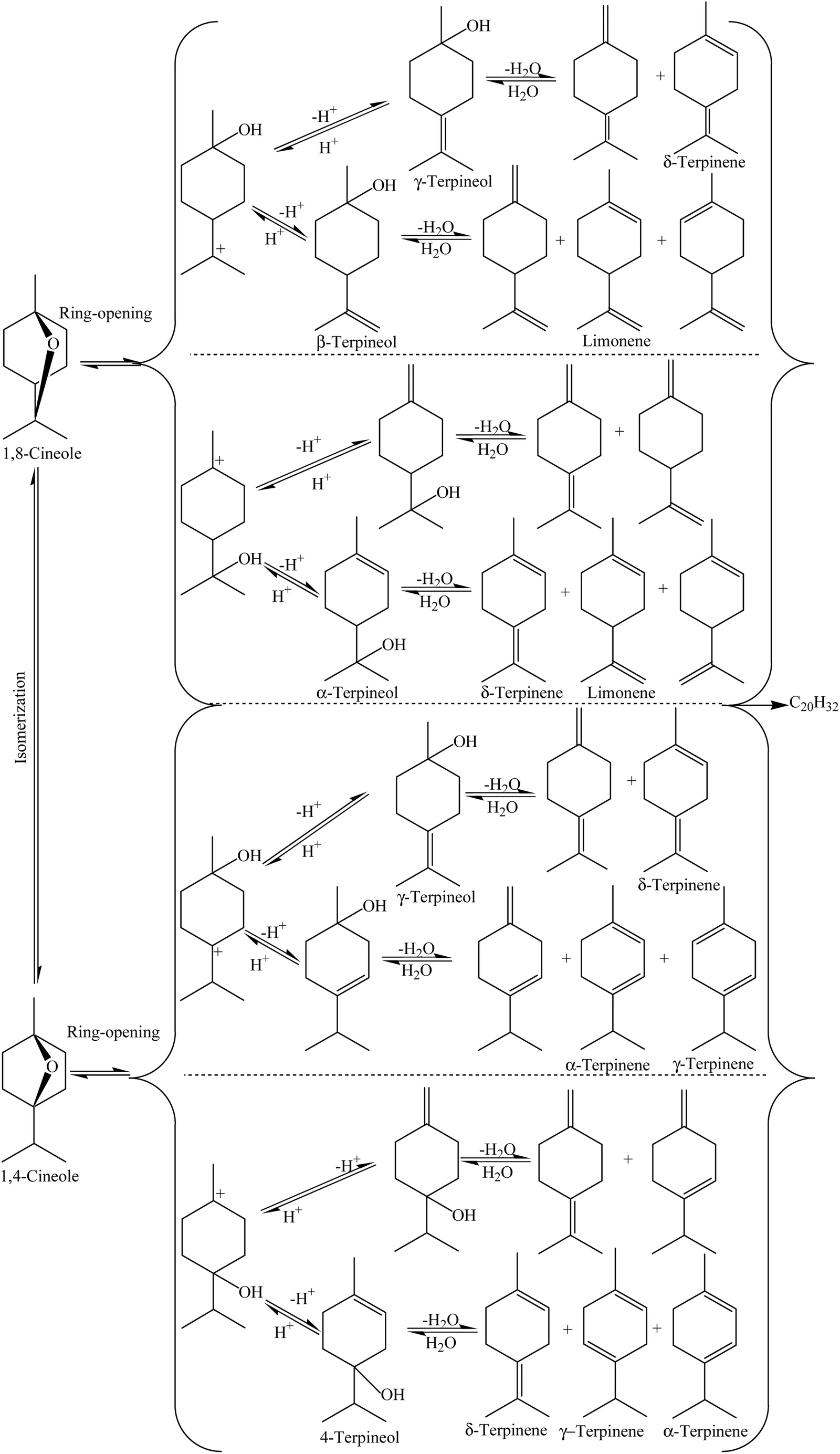
The conversion of 1,8-cineole to terpene dimers.

Ru-supported sulfonic acid-functionalised ionic liquid (RuNPs@SILP-1.00) was investigated for HDO of eucalyptol.^76^ The eucalyptol conversion and alkene/alkane ratio were 44% and 3 : 2, respectively, at 125 °C and 1.0 h. However, complete conversion was achieved at 125 °C and 12 h, which was reduced to 4 h at 150 °C. HDO over unmodified SILP-1.00 resulted in complete conversion, producing *p*-menthadiene selectively *via* ether cleavage and dehydration. The low selectivity of *p*-menthane was observed over Ru nanoparticles stabilized in homogeneous sulfonic acid-functionalized ionic liquid (RuNPs@IL-SO_3_H). It was due to catalyst deactivation owing to the instability of Ru nanoparticles colloids under reaction conditions. HDO of 1,8-cineole was further explored using the combination of various acidic triflates and Pd/C catalysts.^80^ The triflates catalysed the ring-opening reaction to terpinene isomers, which was then converted to a complex mixture of isomers. Adding Pd/C significantly improved the catalytic efficacy and selectivity of *p*-menthanes (*cis* + *trans*) and *p*-cymene. Zr(OTf)_4_ and Hf(OTf)_4_ were more active than lanthanide triflates due to the high charge density of central triflate metal ions.^80^ The *n*-octane solvent improved the conversion to 49.7% due to enhanced heat and mass transfer, accelerated the turnover, minimized adsorption of unreactive molecules, and enhanced hydrogen solubility. The combination of Amberlyst-15 and Pd/C was investigated for 1,8-cineole conversion to *p*-menthane in a one-pot process.^81^*p*-Menthane was the major product, with a small amount of *p*-cymene. The ether was formed as an intermediate dehydration product of 1,8-cineole, which was subsequently converted to *p*-menthane *via* HDO.

### 3.2. Linalool

Linalool, originally produced from rosewood oil, is an unsaturated terpene alcohol commonly found in lavender, coriander, and orange. Recently, it has gained industrial relevance due to its application in the production of vitamin A and E, agrochemicals, and fragrances.^81^ Linalool is commonly synthesized from α-pinene to meet increased demand.^82^ Fermentative linalool production from glucose and glycerol using engineered *E. coli* or yeast emerged as an alternative approach.^83,84^ However, low linalool yield limits the commercialization of this bioprocess. A recent study demonstrated SAF production from linalool using dual heterogeneous catalysts (Table 7).^81^ The process involved solvent-free dehydration, cyclization, and hydrogenation in one pot using Amberlyst-15 and Pd/C catalysts. The two step dehydration and hydrogenation of linalool resulted in isomerized hydrogenated linalool (IHL), comprised ∼60% *p*-menthane, ∼35% 2,6-dimethyloctane (DMO), and ∼4% *p*-cymene (Scheme 6).

**Table 7:** Catalytic conversion of 1,4-cineole, linalool, α-cedrol, and menthol into SAF^a^

Terpenoid	Catalyst	Reactor	Reaction conditions	Conv. (%)	Desired product	Yield (%)	Other (%)	Ref.
1,4-Cineole	Amberlyst-15	Batch	5 g 1,4-Cineole, 0.25 g catalyst, 85 °C, and 24 h	100	Dimers	59	b	77
	Nafion SAC-13	Batch		90	Dimers	1	c	77
	MMT K-10	Batch		85	Dimers	2	d	77
	Amberlyst-15 + Pd/C	Batch	44 g of 1,4-cineole, 4.51 g Amberlyst-15, 0.44 g Pd/C, 500 psi H_2_, 50 °C for overnight, and 100 °C for 5 h	100	*p*-Menthane	100	—	81
Linalool	Amberlyst-15 + Pd/C	Batch (one-pot)	35.32 g linalool, 3.91 g Amberlyst-15, 0.38 g Pd/C, 500 psi H_2_, 20–25 °C overnight, 50 °C for 3 h, 100 °C for 1 h, and 150 °C for 1 h	—	*p*-Menthane	55	DMO (41) and CY (3)	81
	Amberlyst-15 + Pd/C	Batch (two-pot)	Dehydration: 432.5 g linalool, 43.7 g Amberlyst-15, 50 °C, and 1 h. Hydrogenation: 37.41 g dehydration product, 4.05 g Amberlyst-15, 0.37 g Pd/C, 500 psi H_2_, 20–25 °C overnight, 50 °C for 3 h, 100 °C for 3 h, and 150 °C for 1 h	—	*p*-Menthane	60	DMO (35) and CY (4)	81
	2 GH, AlPO_4_/MgSO_4_, PtO_2_	Batch (three-pot)	Ring-closing metathesis: 30 g of linalool, 0.1 mol% catalyst, ambient temperature, and 45 min. Dehydration: 13.01 g 2-methyl cyclopentenol, 1.34 g AlPO_4_/MgSO_4_, 60 °C, 5 h, and 40 torr vacuums. Hydrogenation: PtO_2_ and 40 psi	—	RJ-4	91	—	86
α-Cedrol	PTA and PtO_2_	Batch	Dehydration: 162.4 g of α-cedrol, 1.745 g *p*-toluenesulfonic acid monohydrate, and 4.5 h. Hydrogenation: 450 mL α-cedrene, 225 mL acetic acid, 0.2 g PtO_2_, and 50 psi H_2_	—	α-Cedrane	97	—	91
Menthol	Nb_2_O_5_	Semi-batch	100 mL menthol solution (0.05 mol l^−1^ in cyclohexane), 0.375 g catalyst, 20 bar H_2_, 190 °C, 600 rpm, and 4 h	100	*p*-Menthane	3	*p*-M (97)	93
	Pt/Nb_2_O_5_			100		86	BC (14)	93
	Pd/Nb_2_O_5_			100		88	BC (12)	93
	Ru/Nb_2_O_5_			71		54	*p*-M (34) and BC (12)	93
	Pd/SiO_2_			18		59	BC (41)	93
	Ru/SiO_2_			12		17	*p*-M (54) and BC (29)	93

a BC: branched cycloparaffins, *p*-M: *p*-menthene, DMO: 2,6-dimethyloctane, PTA: *p*-toluenesulfonic acid monohydrate, 2 GH: 2nd generation Grubbs–Hoveyda, and CY: *p*-cymene.

b Terpinolene = 10, α-terpinene = 16, γ-terpinene = 5, *p*-cymene = 5, and limonene = 1.

c 1,8-Cineole = 2, terpinolene = 10, α-terpinene = 51, γ-terpinene = 13, terpinene-1-ol = 4, *p*-cymene = 3, and limonene = 2.

d 1,8-Cineole = 1, terpinolene = 6, α-terpinene = 59, γ-terpinene = 8, terpinene-1-ol = 2, *p*-cymene = 2, and limonene = 1.

**Scheme 6:**
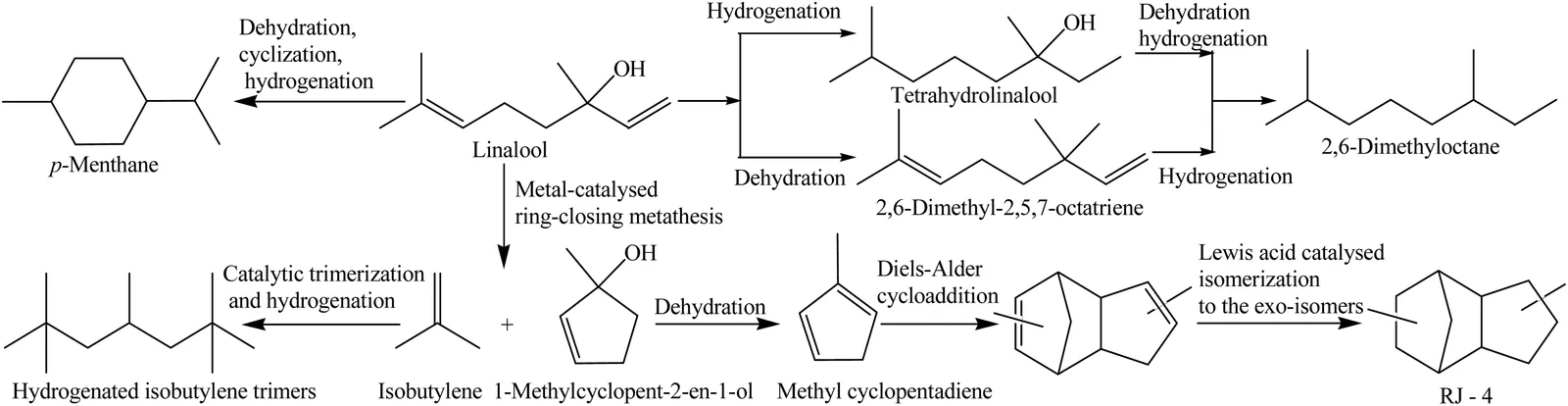
Catalytic conversion of linalool to SAF.

Interestingly, a distinct patented pathway also achieved DMO production from linalool. This route involved linalool hydrogenation to tetrahydrolinalool (THL), followed by its dehydration using Montmorillonite K10 under Dean–Stark conditions, forming a mixture of 3,7-dimethyloctene isomers. These isomers then followed two parallel routes: first, they ensued hydrogenation to yield DMO directly, and second, they underwent acid-catalysed oligomerization to form C_20_ dimers, C_30_ trimers, and C_40+_ oligomers, which were subsequently hydrogenated to saturated hydrocarbons. The final product mixture, referred to as IHL, has 43.54 MJ kg^−1^ heat of combustion, which is 1.7% higher than Jet A.^66,85^ Though the density of IHL was satisfactory for the Jet A, the kinematic viscosity was 2.74 mm^2^ s^−1^ at −20 °C. The viscosity was well below the desired value for the Jet A.^81^ The IHL product mixture leverages the high heat of combustion of DMO, the high density of *p*-menthane, and the low viscosity of both components. However, the volumetric heat of combustion of the IHL remained lower than that of RJ-4. Nevertheless, this product mixture can be blended with linalool-derived RJ-4 or other aviation fuels.

Further studies were conducted to convert linalool into methylcyclopentadiene dimers using Ru-catalysed ring-closing metathesis, followed by acid-catalysed dehydration and dimerization.^86,87^ The second-generation Grubbs–Hoveyda catalyst demonstrated superior catalytic activity, achieving complete conversion of 1-methylcyclopent-2-en-1-ol under ambient conditions with a remarkable turnover number of 4400 at just 0.01 mol% catalyst loading.^86,87^ The reaction was conducted under solvent-free conditions, minimizing environmental impact and energy input. Interestingly, at elevated temperatures (60 °C), these catalysts also promoted partial dehydration of the alcohol to methylcyclopentadiene (MCPD), along with the formation of dimers and oligomers, demonstrating the dual role of the catalyst. To optimize this transformation, several homogeneous and heterogeneous acid catalysts were screened. Strongly acidic Nafion SAC-13 and Montmorillonite K10 achieved high conversions of 1-methylcyclopent-2-en-1-ol, yielding significant quantity of oligomerized products. Whereas, a mixed aluminium phosphate (AlPO_4_) and magnesium sulphate (MgSO_4_) selectively dehydrated 1-methylcyclopent-2-en-1-ol to MCPD and enabled its dimerization to well-defined isomers of methylcyclopentadiene dimers (MCPD-D). The resulting dimers, dominated by 3,9- and 4,9-dimethyl-*endo*-tricyclodecadiene, were further hydrogenated using PtO_2_ to produce RJ-4.^86,87^

A recent study prepared seven different fuel blends using the precursors derived from linalool and isoprene to demonstrate their suitability as SAF.^88^ Particularly, L-Jet blend was obtained using hydrocarbons derived from linalool, including 30% hydrogenated methylcyclopentadiene dimers, 25% *p*-menthane, 25% DMO, and 20% hydrogenated isobutylene trimers. Other blends included mixing Jet A or HEFA-Jet with cyclic hydrogenated isoprene dimers. The 100% SAF exhibited higher specific heat of combustion, better thermal stability, and higher smoke points, indicating lower soot formation than blends with Jet A (Table 8). The 10 : 90 blend of 1,4-dimethyl cyclo-octane (DMCO) and hydrogenated isoprene dimers (HID) with Jet A met all ASTM requirements, suggesting that these precursors are valuable for rapid fuel certification.

**Table 8:** Properties of pure fuels, dimers, hydrogenated dimers, and SAF blends^a^

Pure/blend		*ρ*	QM	QL	*η*		%*H*	Ref.
					−20 °C	−40 °C		
30% RJ-4 + 20% HIBT + 25% *p*-menthane + 25% 2,6-DMO		0.814	43.17	35.14	4.48	8.77	—	88
∼30% DMCO + 70% HEFA		0.780	43.76	—	5.03	10.54	—	88
∼30% HID + 70% HEFA		0.774	43.68	—	4.57	9.20	—	88
∼10% DMCO + 90% Jet A		0.809	43.17	—	4.12	8.08	—	88
∼10% HID + 90% Jet A		0.807	43.12	—	4.02	7.82	—	88
30% HIBT + 30% DMO + 40% RJ-4		0.817	43.17	35.26	5.40	11.01	—	88
35% HIBT + 35% DMO + 30% RJ-4		0.798	43.35	34.61	4.55	8.89	—	88
40% HIBT + 40% DMO + 20% RJ-4		0.781	43.55	33.99	3.90	7.32	—	88
∼20% Pinane + 80% 5-MU		0.7766	43.68	33.92	5.577	12.18	—	103
∼50% JP-10 + 50% DMO		0.8386	42.99	36.05	4.347	8.219	—	103
∼40% RJ-4 + 60% sabinane		0.8554	42.80	36.61	5.344	10.309	—	103
78% Thujane + 21% pinane		0.828	43.36	35.90	3.48	6.12	—	110
76% Tetrahydroabinene + 23% pinane		0.810	43.25	35.05	2.89	4.81	—	110
97% Carane + 3% 1,1,4-trimethylcycloheptane		0.842	43.19	36.38	3.64	6.84	—	110
90% 1,1,4-Trimethylcycloheptane + 4% carane + 6% *p*-menthane		0.806	43.02	34.69	3.40	6.11	—	110

**Terpene dimer fuels**								
	Camphene	0.941	42.063	39.56	—	34.96^b^	11.75	115
	α-Pinene	0.935	42.047	39.29	—	34.68^b^	11.87	115
	β-Pinene	0.932	41.978	39.13	—	35.05^b^	11.97	115
	Limonene	0.914	41.906	38.30	—	25.86^b^	12.21	115

**Hydrogenated dimers/trimers**								
Hydrogenated α-pinene dimers		0.90^c^	—	39.7	26.5^d^	—	—	112
Hydrogenated β-pinene dimers		0.938	—	39.5	—	—	12.12	107, 109 and 116
Hydrogenated isoprene dimers (HID)		0.806	43.34	34.94	3.10	5.45	—	154
Hydrogenated pure dimers		0.93^c^	—	40.4	3869^d^	—	—	112
Hydrogenated turpentine dimers		0.91^c^	—	38.9	26.5^d^	—	—	112
Hydrogenated isobutylene trimers (HIBT)		0.750	43.99	32.99	3.96	7.08	—	88
Hydrogenation product of valencene		0.88	42.92	37.73	50.2	4.4^b^	—	111
Hydrogenation product of premnaspirodiene		0.88	42.83	37.78	42.9	3.8^b^	—	111
Hydrogenation product of b-caryophyllene		0.85	43.54	37.01	60.5	4.1^b^	—	111

**Pure compounds**								
	*p*-Menthane	0.804	43.20	34.72	2.98	5.19	14.4	103 and 154
	2,6-Dimethyloctane (DMO)	0.733	43.98	32.26	2.27	3.83	15.4	103
	1,4-Dimethylcyclooctane (DMCO)	0.827	43.82	36.22	4.17	7.95	—	154 and 155
	Sabinane	0.810	43.25	35.05	2.892	4.809	14.1	103
	α-Pinane	0.860	43.14	37.08	5.771	11.23	13.2	103
	5-Methylundecane (5-MU)	0.755	43.83	33.12	4.845	10.47	15.5	103
	Valencane	0.879	—	37.63	50.24	—	—	108
	Caryophyllane	0.85	—	36.87	60.47	—	—	107–109
	Premnaspirodiane	0.882	—	37.68	42.91	—	—	107–109
	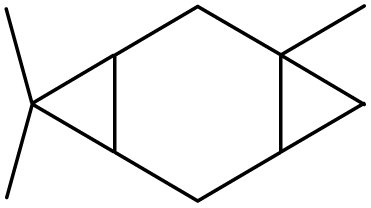	0.884	42.64	37.69	5.60	—	12.30	118
	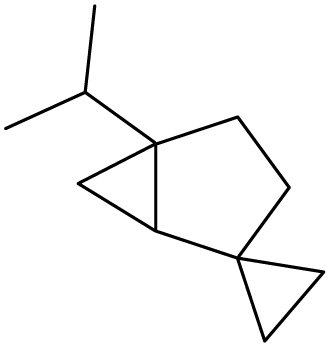	0.870	42.98	37.39	6.21	—	12.05	118
	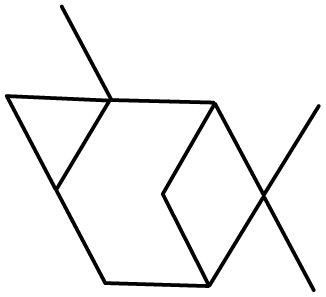	0.900	42.85	38.56	8.18	—	11.99	118
	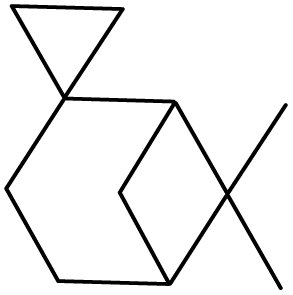	0.896	42.98	38.51	10.64	—	12.24	118
	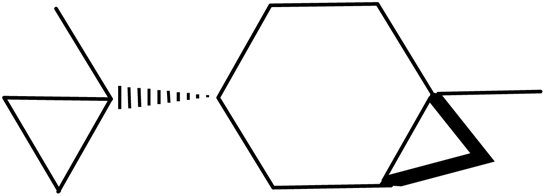	0.880	42.84	37.70	8.81	—	12.43	118
	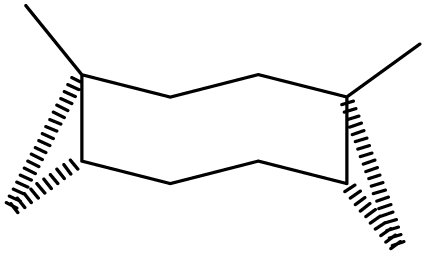	0.892	43.19	38.54	12.87	—	11.94	118
	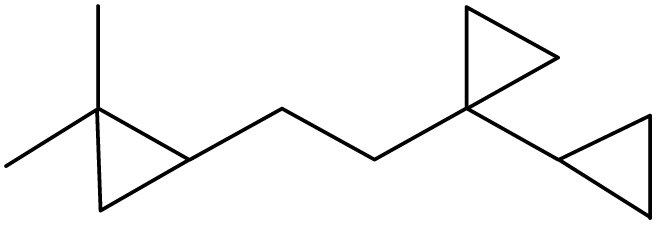	0.8552	43.38	37.10	11.17	—	12.16	118

a *ρ*: density at 15 °C, g mL^−1^, *η*: viscosity, mm^2^ s^−1^, QL: net heat of combustion per litre, MJ L^−1^, and QM: net heat of combustion per kg, MJ kg^−1^.

b At 40 °C.

c At 20 °C.

d At −10 °C.

### 3.3. Cedrol

Cedrol is a sesquiterpene alcohol present in cedar and other essential oils with woody, leather-like, and masculine fragrances. It finds applications in perfumery and pharmaceuticals. *Epi*-cedrol is a potential precursor to cedrenes and can be produced from glucose using metabolically engineered yeasts.^89^ Recently, the dehydration of α-cedrol to α-cedrene was performed using *p*-toluenesulfonic acid (PTA) monohydrate as a catalyst under constant reflux conditions with benzene (Scheme 7 and Table 7).^90,91^ The α-cedrene was subsequently hydrogenated to α-cedrane over PtO_2_ under acidic conditions. The cedrane showed a NHOC of 141.6 kBtu gal^−1^ and 0.923 g mL^−1^ density. Cedrane demonstrated higher density and reduced viscosity, which were close to or superior to JP-10. However, the cetane number (18) of cedrane was below that of RJ-4 and JP-10. To obtain hydrocarbons with higher density and NHOC, isomerization of cedrane was performed over AlCl_3_ to obtain a mixture of alkyl adamantanes, including 1-ethyl-3,5,7-trimethyladamantane mixture (ETMA). However, the ETMA mixture showed only 0.896 g mL^−1^ density and 135.6 kBtu gal^−1^ NHOC. The ETMA mixture also exhibited low viscosity and high cetane numbers, making it suitable for diesel engines.^90,91^

**Scheme 7:**
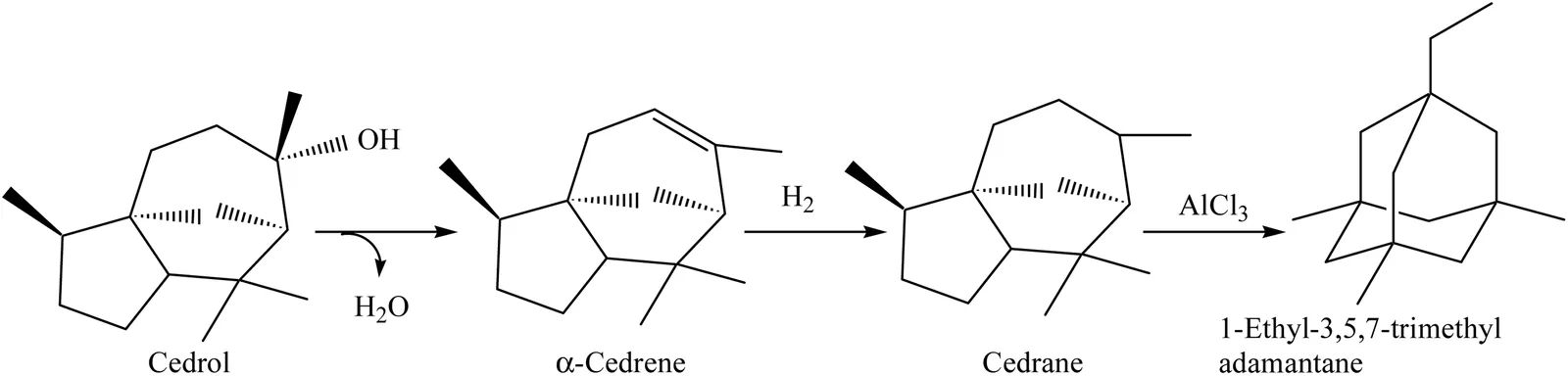
Conversion of α-cedrol to 1-ethyl-3,5,7-trimethyl adamantane (ETMA).

### 3.4. Menthol

Menthol is a cyclic terpenoid and is typically obtained from peppermint and other mint oils. It finds diverse applications in throat lozenges, cough drops, and tobacco products due to its cooling and refreshing sensation. *Mentha arvensis* (mint) is the most common bioresource for menthol.^92^ Menthol can be converted to *p*-menthane by dehydration, followed by *p*-menthene hydrogenation. The one-pot liquid-phase deoxygenation of menthol was thus investigated in the presence of hydrogen over noble metal-supported Nb_2_O_5_ and SiO_2_ catalysts.^93^ The Nb_2_O_5_, with Lewis and Brønsted acidic sites, demonstrated high catalytic activity for HDO, achieving complete menthol conversion. However, *p*-menthane selectivity was barely 3%, with *p*-menthene being the major product due to the absence of metallic sites for hydrogenation (Scheme 8). Conversely, *p*-menthane was the major product over Pt, Pd, and Ru-supported Nb_2_O_5_ catalysts. Among these, Pt and Pd-supported Nb_2_O_5_ exhibited excellent catalytic activity due to synergy between acidic and metal sites, achieving complete conversion of menthol and 86–88% *p*-menthane selectivity.^93^ However, Pd and Ru supported on SiO_2_ exhibited poor catalytic activity due to the weaker acidity of SiO_2_ compared to Nb_2_O_5_.^93^

**Scheme 8:**
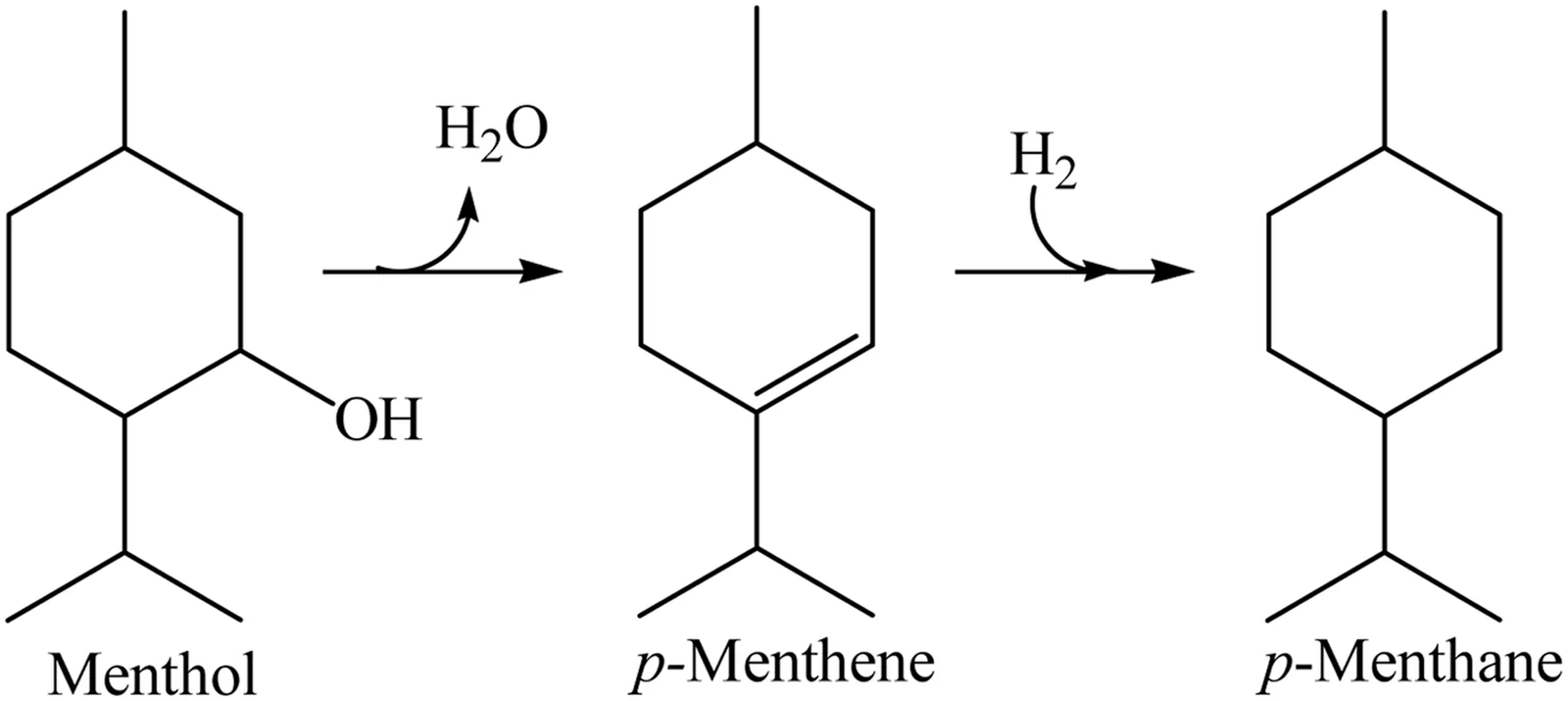
Catalytic conversion of menthol to *p*-menthane.

### 3.5. Nerolidol

Nerolidol is an acyclic sesquiterpene alcohol and is commonly extracted as essential oils from plants such as neroli, ginger, jasmine, lavender, tea tree, *Brassavola nodosa*, and *Cannabis sativa*. It is used in perfumes, cosmetics, and food flavouring, and has therapeutic applications. Nerolidol can also be produced from biomass sugars *via* fermentation.^94,95^ The nerolidol conversion into SAF involved ring-closing metathesis using Grubbs-Hoveyda M720 catalyst (Scheme 9).^96^ A complete conversion was achieved in 20 min at low catalyst loading (0.03 mol%) under solvent-free conditions, yielding an equimolar amount of 1-methylcyclopent-2-en-1-ol (MCP) and 2,6-dimethyl-1,5-heptadiene (DMH). Subsequently, MCP was dehydrated using a weak Lewis acid (AlPO_4_) and a desiccant (MgSO_4_) to produce 2-methylcyclopentadiene, which ensued Diels–Alder cycloaddition over Amberlyst-15 to form dimethyldicyclopentadienes and 2,6-dimethyl-1,5-heptadiene. These intermediates were then hydrogenated over Pd/C to obtain tetrahydrodimethyldicyclopentadiene isomers and 2,6-dimethylheptane (DMH), respectively. The resulting equimolar mixture, designated F1, showed a density of 0.783 g mL^−1^ at 15 °C, well below Jet A, and a kinematic viscosity at −20 °C nearly 60% lower than Jet A, while NHOC was comparable. They observed the partial conversion of 2,6-dimethyl-1,5-heptadiene to the trimethylcyclohexene intermediate, which was subsequently hydrogenated to 1,1,3-trimethylcyclohexane (TMC). A second blend, F2, consisting of a 70 : 30 DMH and TMC molar mixture, demonstrated a 2.1% higher specific NHOC and 70% lower kinematic viscosity at −20 °C compared to Jet A. However, its 1.4% higher density brings F2 to the range of paraffinic kerosene and is certified as a blend for aviation fuel.

**Scheme 9:**
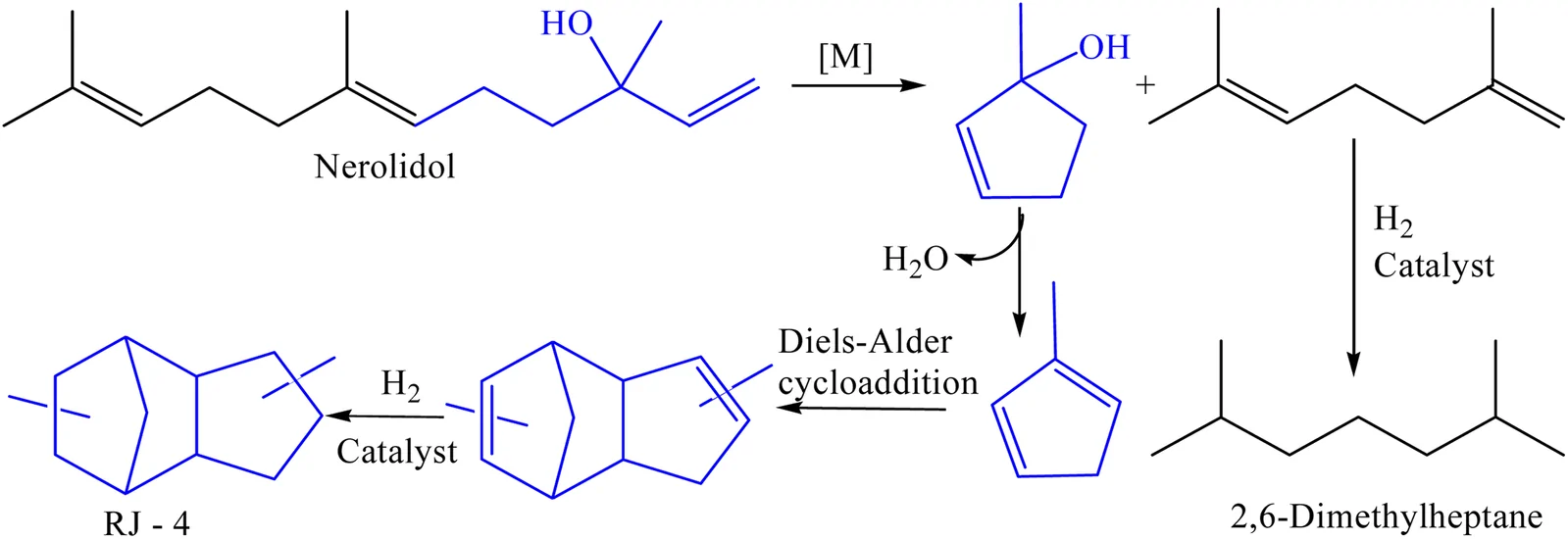
Catalytic conversion of nerolidol to RJ-4.

### 3.6. Geranyl acetone

The geranyl acetone is one of the major compounds found in the essential oil of *Ononis natrix*, *Conyza bonariensis* L., and *Equisetum arvense* L.^97–100^ It is well known for its taste, scent, and beneficial biological properties. In contrast to these food and pharmaceutical uses, catalytic hydroprocessing of geranyl acetone yielded a jet fuel blend.^101^ The Pt/Al_2_O_3_ catalyst exhibited mild acidity and remained active in HDO and condensation reactions. On the contrary, the diffusion properties and acidity were altered by using mesocellular silica foam (MCF) as a support, with the acidity altered by grafting Al onto pure MCF. The Pt/MCF showed acidity, allowing partial deoxygenation of geranyl acetone. However, complete deoxygenation was attained over Pt/Al_2_O_3_ and Pt/Al-MCF-20 due to superior acidity compared to the Pt/MCF. The Pt/Al-MCF-20 showed higher stability up to 100 h, while the Pt/MCF deactivated due to aggregation of Pt particles.^101^ The hydrogenated product of geranyl acetone meets the ASTM standards. The density, freezing point, flash point, heat of combustion, and aromatic content of the hydrogenated products were 0.80 g mL^−1^, −72 °C, 51 °C, 45 MJ kg^−1^, and <1%, respectively.

## 4. Pure terpenes to SAF

Cyclic terpenes such as α-pinene, limonene, and sabinene are generally harvested from natural sources. They can also be produced biologically from biomass-derived glucose using metabolically engineered microorganisms with moderate titters, though this approach is not yet commercially viable.^102^ Their cyclic structure can be leveraged for producing high-density SAF. These terpenes are converted to SAF-range cycloparaffins through hydrogenation, isomerization, dimerization, cyclopropanation, or their combinations, as described below.

### 4.1 Hydrogenation

The oxygen and double bonds affect fuel stability and energy density. Hydrogenation is thus used to saturate terpene double bonds, thereby improving fuel properties. Hydrogenation of α-pinene, limonene, and sabinene was examined using PtO_2_ and Pd/C catalysts to produce cycloparaffin-rich SAF blend stocks (Scheme 10 and Table 9).^103^ Hydrogenation of α-pinene and limonene over Pd/C resulted in pinane-containing blends of two diastereomers and dehydrogenation product, *p*-cymene, respectively. However, PtO_2_ significantly reduced double-bond isomerization and selectively produced *p*-menthane. The sabinene mainly produced a mixture of diastereomers of tetrahydrosabinene and pinane. Densities of pure fuels at 15 °C ranged from 0.733 to 0.939 g mL^−1^. JP-10 and RJ-4 showed the highest density, followed by bicyclic pinane, monocyclic sabinene and *p*-menthane, and acyclic SAF HEFA, 5-methylundecane (5-MU), and 2,6-dimethyloctane (DMO) (Table 8). RJ-4 and JP-10 exhibited the highest viscosities, followed by pinane, sabinene, and *p*-menthane. The study was further extended to demonstrate the suitability of blending these saturated terpenes and military aviation fuels (JP-10 and RJ-4) with synthetic paraffinic kerosene (SPK). The cycloparaffins, JP-10, and RJ-4 were blended with SPKs like HEFA-Jet, 5-MU, and DMO, whose density and NHOC were lower. All blends were optimized to have density above 0.775 g mL^−1^ and kinematic viscosity below 12.0 mm^2^ s^−1^ at −40 °C.^103^ The blending of 40–50% hydrogenated monoterpenes with SPKs exceeded the performance of Jet A. The highest specific NHOC was 43.68 MJ kg^−1^ for 20% pinane-5-MU blend, and the highest volumetric NHOC was 36.05 MJ L^−1^ in the case of an equal blend of JP-10 and DMO, surpassing the minimum requirements of Jet A by 2.1% and 8.7%, respectively. These SAFs are free of aromatics and offer lower emissions of particulates and harmful byproducts. However, further research is needed on emissions, material compatibility, and scaling up biological production of monoterpenes for commercial use.^103^

**Scheme 10:**
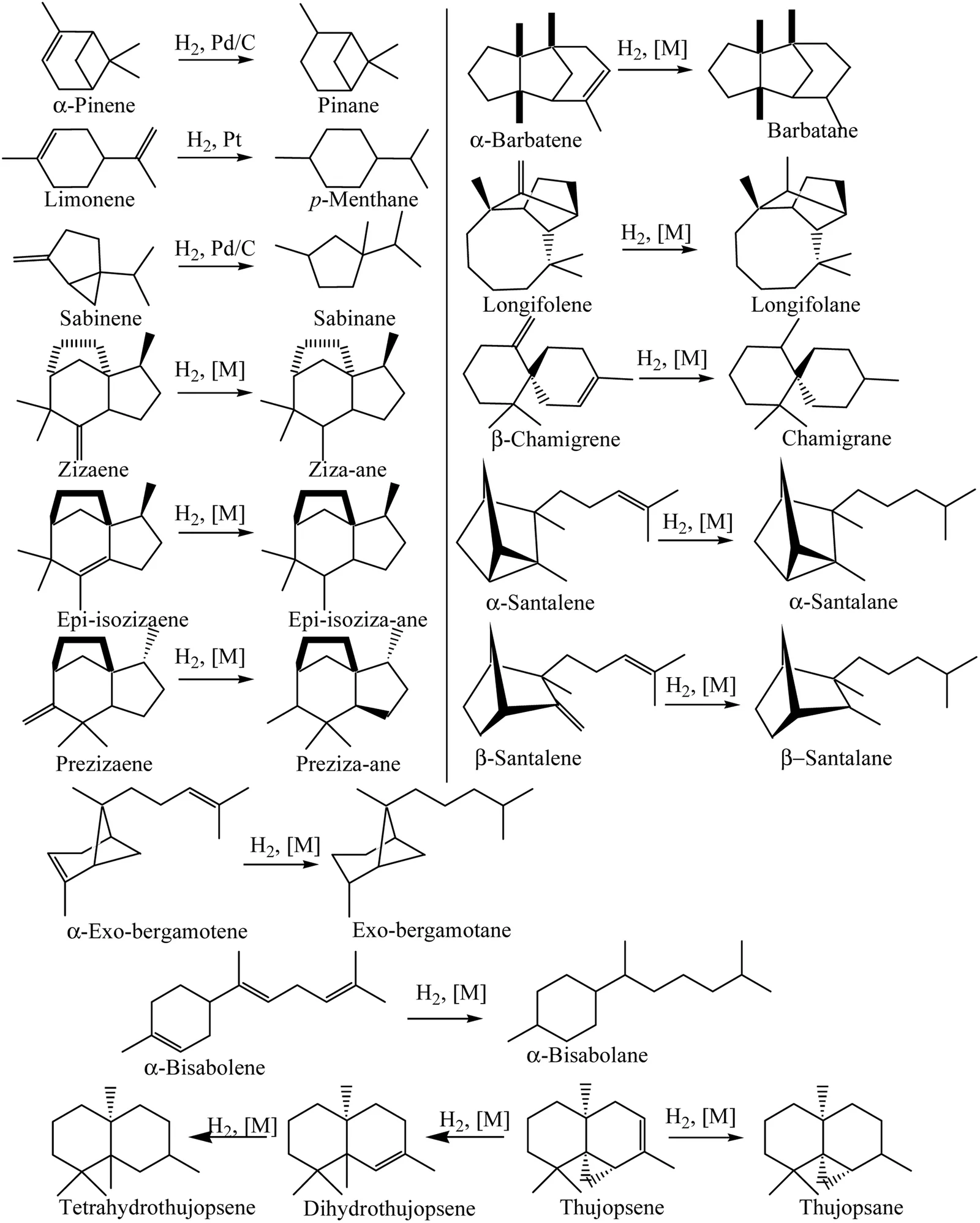
Hydrogenation of cyclic terpenes (α-pinene, limonene, and sabinene).

**Table 9:** One-pot catalytic conversion of terpenes to SAF in a batch reactor^a^

Terpene	Catalyst	Reaction conditions	Major product (%)	Fuel properties					Ref.
				*ρ*	QL	QM	*η*	%*H*	
α-Pinene	Pd/C	51.14 g α-pinene, 0.5 g catalyst, 10 mL glacial acetic acid, ambient rtemperature, 24 h, and 276 kPa H_2_	α-Pinane (100%)	0.860	37.08	43.14	11.23	13.2	103
	ZnCu/CH_2_Br_2_	35.10 g α-pinene in 80 mL ether, 50 mL dibromo methane in 50 mL ether, 50 °C, and 18 h	2,7,7-Trimethyl tricyclo[4.1.1.02,4]octane (45.1%)	0.900	38.56	42.85	8.18^h^	11.99	118
β-Pinene	ZnCu/CH_2_Br_2_	18.58 g β-pinene in 40 mL ether, 25 mL dibromomethane in 24 mL ether, 50 °C, and 18 h	6,6-Dimethyl spiro[bicyclo [3.1.1]heptane-2,1′-cyclo propane] (69.6%)	0.896	38.51	42.98	10.64^h^	12.24	118
Limonene	PtO_2_	49.85 g limonene, 0.5 g catalyst, 10 mL glacial acetic acid, ambient temperature, 24 h, and 276 kPa H_2_	*p*-Menthane^b^	0.804	34.72	43.20	5.19	14.4	103
	ZnCu/CH_2_Br_2_	36.38 g limonene in 80 mL ether, 48 mL dibromo methane in 40 mL ether, 50 °C, and 18 h	1-Methyl-4-(1-methyl cyclopropyl)bicyclo [4.1.0]heptane (56.2%)	0.880	37.70	42.84	8.81^h^	12.43	118
Sabinene	Pd/C	51.62 g sabinene, 0.5 g catalyst, 10 mL glacial acetic acid, ambient temperature, 24 h, and 276 kPa H_2_	Tetrahydrosabinene diastereomers (76%)^c^	0.810	35.05	43.25	4.809	14.1	103 and 110
	PtO_2_	16.09 g sabinene, 0.21 g catalyst, 4 mL glacial acetic acid, 17–19 °C, 50 psi H_2_, and 4 h	Thujane (75%)^d^	0.828	35.90	43.36	6.12	—	110
	ZnCu/CH_2_Br_2_	17.31 g sabinene in 40 mL ether, 26 mL dibromo methane in 20 mL ether, 150 °C, and 18 h	5-Isopropyl spiro[bicyclo[3.1.0]hexane-2,1′-cyclopropane] (59.7%)	0.870	37.39	42.98	6.21^h^	12.05	118
3-Carene	Pd/C	12.57 g 3-carene, 0.2 g catalyst, 2 mL glacial aceticacid, 17–19 °C, 500 psi H_2_, 150 °C, and 24 h	1,1,4-Trimethyl-cycloheptane (90.0%)^e^	0.806	34.69	43.02	6.11	—	110
	PtO_2_	48.1 g 3-carene, 0.55 g catalyst, 1 mL glacial acetic acid, 17–19 °C, 40 psi H_2_, ambient temperature, and 24 h	Carane diastereomers (97%)^f^	0.842	36.38	43.19	6.84	—	110
	ZnCu/CH_2_Br_2_	34.56 g 3-carene in 80 mL ether, 50 mL dibromo methane in 45 mL ether, 50 °C, and 18 h	1,4,4-Trimethyl tricyclo[5.1.0.0]octane (76.4%)	0.884	37.69	42.64	5.60^h^	12.30	118
1,6-Dimethyl-1,5-cyclooctadiene	ZnCu/CH_2_Br_2_	11.14 g 1,6-dimethyl-1,5-cyclooctadiene in 40 mL ether, 24.5 mL dibromo methane in 25 mL ether, 50 °C, and 18 h	1,4-Dimethyl tricyclo[7.1.0.04,6]decane (75.4%)	0.892	38.54	43.19	12.87^h^	11.94	118
Myrcene	ZnCu/CH_2_Br_2_	10 g myrcene in 60 mL ether, 20 mL dibromo methane in 20 mL ether, 50 °C, and 18 h	1-(2-(2,2-Dimethyl cyclopropyl)ethyl)-1,1′-bi(cyclopropane) (20%)	0.8552	37.10	43.38	11.17^h^	12.16	118
	CF_3_COOZnCH_2_I	5 mmol myrcene in 7.5 mL CH_2_Cl_2_, 25 °C, and 6 h	Tricyclic propane (69.8%)^g^	0.85	—	43.4	10.3^h^	—	119

a *ρ* = density at 15 °C, g mL^−1^, *η* = viscosity at −40 °C, mm^2^ s^−1^, QL = net heat of combustion per litre, MJ L^−1^, and QM = net heat of combustion per kg, MJ kg^−1^.

b *p*-Cymene (∼2%).

c Pinane diastereomers (23%).

d Pinane diastereomers (23%) and tetrahydrosabinene (2%).

e *p*-Methane (6%) and carane (4%).

f 1,1,4-Dimethyl cycloheptane (3%).

g Bicyclic propane (20%) and monocyclic propane (10.2%).

h At −20 °C.

The U.S. Navy patented a technology for producing high-density fuels from sesquiterpenes such as bisabolene, α-barbatene, β-chamigrene, zizaenes, longifolene, santalenes, and thujopsene, sourced through fermentation or extracted from natural sources.^104–109^ These sesquiterpenes were hydrogenated and isomerized using Brønsted or Lewis acid catalysts (Nafion SAC-13 and zeolites), then further hydrogenated over Pd, Pt, or Ni catalysts. The resulting fuels exhibited volumetric NHOC of 138–140 kBtu gal^−1^ and 0.81–0.94 g mL^−1^ density, surpassing the energy content of JP-10. Particularly, barbatene/thujopsene-based fuels matched or outperformed JP-10 in terms of NHOC. In some embodiments, diamondoid hydrocarbons (1-ethyl-3,5,7-trimethyladamantane) were produced, further enhancing volumetric energy density and oxidative stability. However, some of these fuels exhibited higher low-temperature viscosity, limiting their standalone application in aviation engines, but these issues could be mitigated through blending with SPKs.^104–109^

Sabinene and 3-carene, featuring cyclopropane rings, were chemo-selectively hydrogenated using reduced PtO_2_ and Pd/C (Table 9).^110^ Hydrogenation over Pd/C yielded the *cis*–*trans* mixture of thujane and carane from sabinene and 3-carene, respectively, preserving the ring structures. In contrast, Pd/C demonstrated complete hydrogenation of sabinene and 3-carene to tetrahydrosabinene and 1,1,4-trimethylcycloheptane, respectively. Thujane and carane exhibited higher densities than the completely hydrogenated products due to hydrogenation of the cyclopropane ring (Table 8). These hydrogenated monoterpenes showed 4.0–8.6% higher density, 0.5–1.3% greater specific NHOC, and significantly lower viscosity than aviation fuels.

High-density SAF was obtained *via* biological production of sesquiterpenes such as valencene, premnaspirodiene, and β-caryophyllene through metabolic engineering, followed by hydrogenation and isomerization over Pd and Pt catalysts.^111^ These hydrocarbons showed significantly higher density and up to 13% higher volumetric NHOC (133–141 kBtu gal^−1^) compared to aviation fuels, though they exhibited higher low-temperature viscosity. Premnaspirodiene, due to its spiro-linkage, demonstrated a higher cetane number and the lowest viscosity among the sesquiterpenes, making it more suitable for diesel blending but incompatible as a standalone fuel.^111^ Isomerization of sesquiterpenes with Nafion SAC-13 produced a mixture of isomers, primarily δ-selinene. However, isomerizing valencene and premnaspirodiene did not yield structures for improving fuel properties but resulted in the formal synthesis of δ-selinene.

### 4.2 Isomerization and dimerization

Isomerization and dimerization of α-pinene into energy-dense SAF were studied using a heteropoly catalyst, Mn_0.5_[MIMPS]_2_PW_12_O_40_, comprising sulfonated organic cations and manganese ions, responsible for the Brønsted and Lewis acidity, respectively (Scheme 11 and Table 10).^112^ It promoted both isomerization and dimerization of α-pinene, achieving 100% conversion and yielding a mixture of approximately 60% dimers and 40% isomers. This hydrocarbon mixture, following hydrogenation, exhibited impressive fuel properties, such as high density (0.90 g mL^−1^ at 20 °C), low freezing point (−66 °C), and high volumetric NHOC (39.7 MJ L^−1^), comparable to JP-10 (Table 8). Another study demonstrated selective dimerization of β-pinene using Montmorillonite K10 (MMT-K10) and Nafion, yielding approximately 75% and 90% dimer, respectively.^113^ However, byproduct *p*-cymene was also observed over MMT-K10 due to its dual (Lewis and Brønsted) acidity. Nafion, despite its lower surface area, achieved higher selectivity at around 100 °C. The hydrogenated dimers showed a density of 0.938 g mL^−1^ and a volumetric NHOC of 39.5 MJ L^−1^, equivalent to JP-10.

**Scheme 11:**
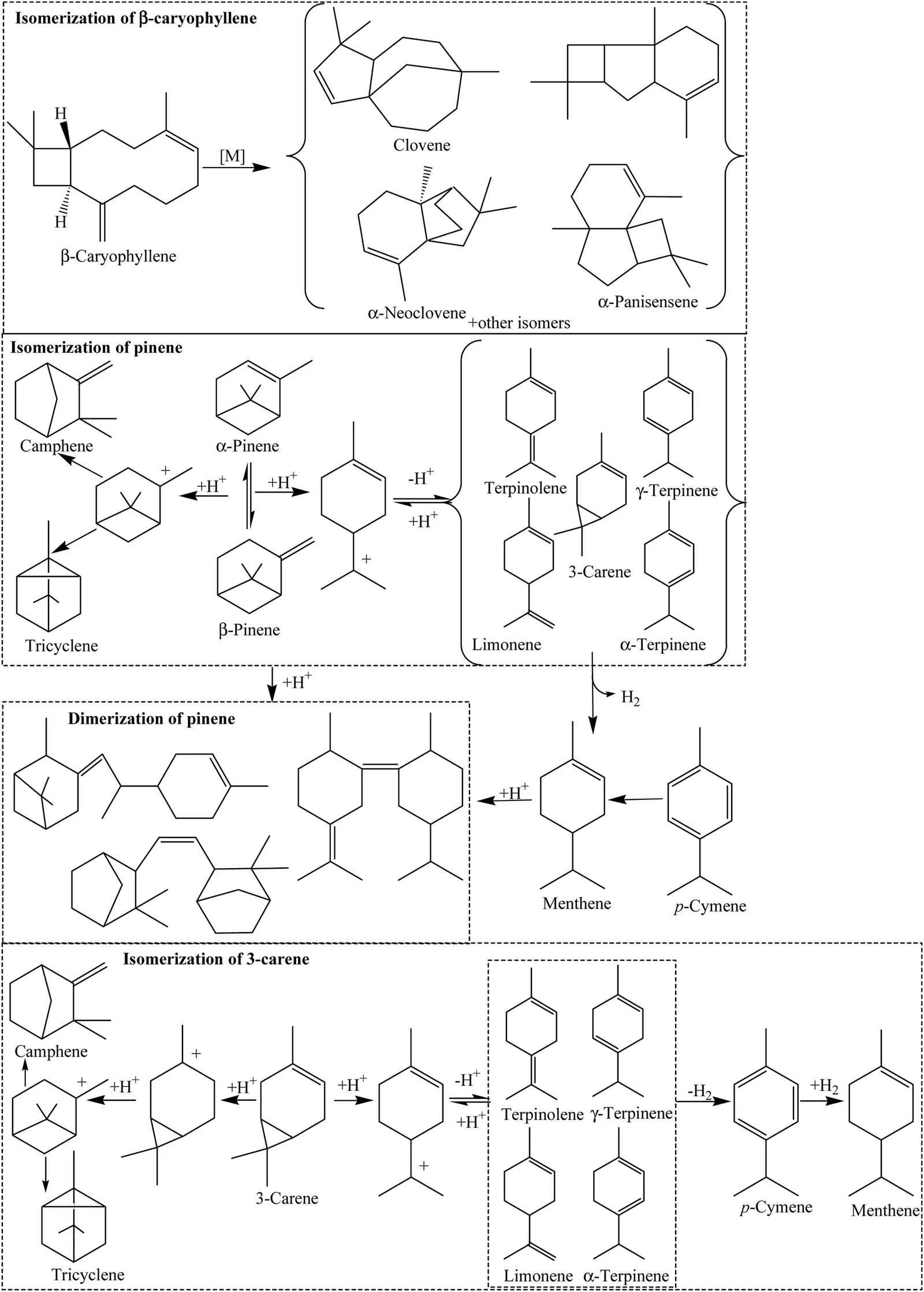
β-Caryophyllene, pinenes, and 3-carene isomerization and pinene dimerization.

**Table 10:** Two-step catalytic conversion of terpenes to SAF^a^

Terpene	Catalyst	Reactor	Reaction conditions	Main product (%)	Byproduct (%)	Fuel properties					Ref.
						*ρ*	QL	QM	*η*	%*H*	
β-Pinene	MMT K-10	Flow and batch	Dimerization: 35 g β-pinene, 0.5 g catalyst, 10 mL *n*-heptane, 100 °C, and 1 h, and N_2_ atmosphere. Hydrogenation: 35 mg PtO_2_, 100 mL dimer, 1–2 psi H_2_, and 12–24 h	Dimers (76%)	Isomers (24%)	0.932	39.12	41.978	35.05	11.97	113,115
	Nafion	Flow	Dimerization: 35 g β-pinene, 100 mg catalyst, 10 mL *n*-heptane, 100 °C, 2 h, and N_2_ atmosphere. Hydrogenation: 1 wt% PtO_2_, 1–2 psig H_2_, 12–24 h. OR 100 mg PtO_2_, 100 mL dimer, 30–40 psi H_2_, and 2 to 5 h	Dimers (>90%)	Isomers (∼10%)	0.938	39.5	42.118	—	12.12	113 and 114
α-Pinene	Nafion	Flow and batch	Dimerization: 100 mL α-pinene, 500 mg catalyst, 30 mL *n*-heptane, 100 °C, and 18 h. Hydrogenation: 100 mg PtO_2_, 100 mL dimer, 30–40 psi H_2_, and 2–5 h	Dimers (79%)	Monomers (7%), trimers (14%)	0.935	39.31	42.047	34.68	11.87	114
	Mn_0.5_ [MIMPS]_2_ PW_12_O_40_	Batch	Dimerization: 8.6 g α-pinene, 0.6 g catalyst, 10 mL toluene, 80 °C, 600 rpm, and 16 h. Hydrogenation: 2 wt% Pd/C, 3 Mpa H_2_, 160 °C, and 3 h	Dimers	b	0.9	39.7	—	26.5^c^	—	112
Limonene	Nafion SAC-13	Flow and batch	Dimerization: 100 mL limonene, 40 mL heptane, 1.5 g catalyst, 100 °C, and 2 h. Hydrogenation:100 mg PtO_2_, 100 mL dimer, 30–40 psi H_2_, and 2–5 h	Dimers (85%)	Monomers (15%)	0.914	38.30	41.906	25.86	12.21	114
Camphene	Nafion	Flow and batch	Dimerization:95 g camphene, 500 mg catalyst, 40 mL heptane, 100 °C, and 20 h. Hydrogenation: 100 mg PtO_2_, 100 mL dimer, 30–40 psi H_2_, and 2–5 h	Dimers (86%)	Trimers (14%)	0.941	39.581	42.063	34.96	11.75	114
α-Cedrol	PTA and PiO_2_	Batch	Dehydration: 162.4 g α-cedrol, 1.745 g catalyst, and 4.5 h. Hydrogenation: 450 ml α-cedrene, 225 ml acetic acid, 0.2 g PtO_2_, and 50 psi H_2_	a-Cedrane (>97%)	—	0.923	39.46	—	4.8	—	91

a *ρ* = density at 15 °C, g mL^−1^, *η* = viscosity at 40 °C, mm^2^ s^−1^, QL = net heat of combustion per litre, MJ L^−1^, QM = net heat of combustion per kg, MJ kg^−1^, and PTA = *p*-toluenesulfonic acid monohydrate.

b Camphene, limonene, α-terpinene, d-terpinolene, γ-terpinene, menthane, *p*-cymene, tricyclene, menthene, 3-carene, β-pinene, and others.

c At −10 °C.

Dimerization of pure (pinene, limonene, and camphene) and mixed (crude turpentine) terpene was studied to produce high-density SAF (Table 10).^114–116^ Although Nafion SAC-13 and Nafion-silica nanocomposite dimerized limonene with 85% conversion to dimer, the α-pinene, β-pinene, and camphene remained unreacted. However, Nafion dimerized both α-pinene and camphene, with 79% and 86% yields, respectively. The reaction followed isomerization, particularly for β-pinene and α-pinene, into a mixture, such as camphene and limonene, followed by dimerization. The resulting dimers exhibited similar specific NHOC (around 42 MJ kg^−1^), although volumetric NHOC varied with density. Camphene-derived dimers exhibited the highest volumetric NHOC (142 kBtu gal^−1^), making them comparable to JP-10.^114,115^ Dimerization of crude turpentine yielded 81% dimers with 7% trimer over Nafion SAC-13 and 66% dimer with 11% trimer over MMT-K10. The study also explored the effect of α-pinene/camphene ratios on dimerization yield, with high dimer conversion (average 87%) over Nafion SAC-13 across various compositions.^114^

Turpentine dimers showed a high density and volumetric NHOC, making it ideal for military and aviation fuels, though their high viscosity at low temperatures limited standalone applicability.^117^ These terpene dimers were blended with JP-10, JP-8, RJ-4, and RJ-5. JP-8 blends significantly reduced viscosity while maintaining high density and improved NHOC. Among pinane, decalin, and decane blends with turpentine dimers, decane showed viscosity nearly identical to the JP-8 blend. They also measured the glass transition and melting points of various fuel blends, demonstrating their low-temperature performance, with JP-8 particularly effective in lowering viscosity and enhancing flow properties.^117^

### 4.3 Cyclopropanation

The acid-catalysed dimerization of terpenes is a potential approach to obtain high-density SAF with elevated volumetric NHOC.^79,114^ However, the high viscosity of the resulting C_20_ hydrocarbons limited their application for blending only.^117^ Recently, cyclopropanation has been reported as an alternate approach to produce C_11_–C_13_ alkane-range high-density SAF with elevated volumetric NHOC while maintaining moderate viscosity by controlling molecular weight (Scheme 12).^117^ The cyclopropanation of 3-carene, sabinene, α-pinene, β-pinene, limonene, 1,6-dimethyl-1,5-cyclooctadiene, and myrcene was carried out in a batch reactor.^118^ Cyclopropanation introduces ring strain, with 10–16% higher density than Jet A and 16% more volumetric NHOC than aviation fuels. The cyclopropanated products exhibited lower specific NHOCs (0.03–0.99%) than the starting monoterpenes. Low-temperature viscosity ranged from 5.60–12.87 mm^2^ s^−1^ (Table 9).

**Scheme 12:**
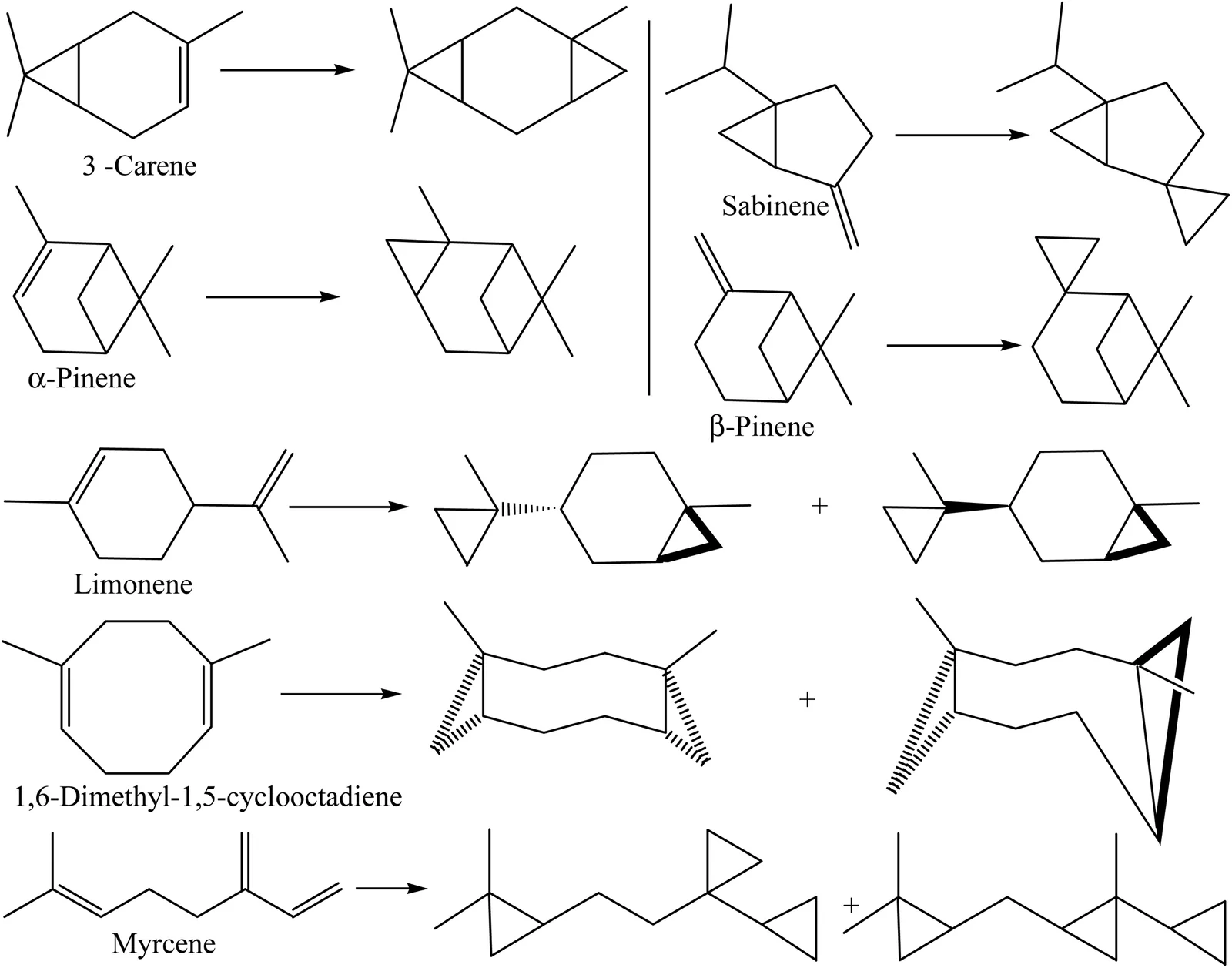
Cyclopropanation of 3-carene, sabinene, α-pinene, β-pinene, limonene, 1,6-dimethyl-1,5-cyclooctadiene, and myrcene.

Structural variation in monoterpenes influences fuel properties. Bicyclic monoterpenes like α- and β-pinene showed the highest density and energy potential, while monocyclic compounds like limonene displayed moderate viscosity and density. Compounds with the spiro linkage exhibited higher density and viscosity than those with the fused-ring system. No glass transition temperatures or freezing points were observed during the low-temperature property studies. These fuels achieved specific impulse (*I*_sp_) values up to 1.2% higher than Rocket Propellant-1 (RP-1) and density-specific impulse (density-*I*_sp_) increases of up to 2.6–4.1%, which could enable greater payloads and reduced tank sizes.^118^ Heat of formation was calculated using the G4 composite method, revealing that Δ*H*_f_ values ranged from (−27.5) − (+60.30) kJ mol^−1^, which were substantially more endothermic compared to JP-10 (−105.5 kJ mol^−1^) and RP-1 (−310.8 kJ mol^−1^). Despite these promising results, the cost-effectiveness and scalability of cyclopropanation remained a challenge due to the use of halogenated reagents.^118^ Myrcene is a naturally derived triolefin and can be derived from the thermal decomposition of β-pinene. The cyclopropanation of myrcene into three-membered rings was carried out using zinc carbenoid reagent under a nitrogen atmosphere.^11,119^ Nearly 100% myrcene conversion and 69.8% yield of tricyclic product were achieved under optimum conditions: 1 : 3 molar ratios of myrcene to zinc carbenoid at 25 °C for 6 h.^119^

Multicyclic sesquiterpenes (pinane, limonene dimer, and turpentine dimer) are derived from biosynthetic pathways and contribute to high density and NHOC. However, they have low cetane numbers and high viscosities, limiting their fuel performance. The sesquiterpenes and 5-methyl undecane were thus blended to overcome these issues. The 5-methyl-undecane, a SPK derived from 1-hexene, exhibited a high cetane number and lower viscosity, with improved ignition and flow characteristics at low temperatures.^79^ The blend with 65% sesquiterpenes and 35% 5-methyl-undecane achieved 45.7 cetane number, 0.853 g mL^−1^ density, and 134 kBtu gal^−1^ NHOC, making it suitable as a diesel fuel surrogate.^120^ In contrast, 40% sesquiterpenes and 60% SPK blend exhibited 57 cetane number and 0.806 g mL^−1^ density, with kinematic viscosity low enough for use at −20 °C, aligning with SAF specifications. This approach demonstrated that careful blending of bioderived compounds could surpass the energy density of aviation fuels by up to 13%, meeting military and commercial application standards.

## 5. Plant oil to SAF

Conversion of pure terpenes and terpenoids generally produces a single hydrocarbon that does not meet SAF specifications, including carbon chain length. In contrast, essential oils contain a variety of these compounds, making them suitable for producing mixed components with better fuel properties, as discussed below (Table 4).

### 5.1. Cedarwood oil

Cedarwood oil is a mixture of multicyclic sesquiterpenes, such as thujopsene (51%), α-cedrene (32%), β-cedrene (7%), and minute quantities of other sesquiterpenes, which vary depending on its origin and climate conditions.^91^ Hydrogenation of cedarwood oil was performed in acetic acid with colloidal Pt at a hydrogen pressure of 400–440 psi (Table 11).^91^ Hydrogenation of α-cedrene and β-cedrene yielded a pair of enantiomers. However, conversion of thujopsene ensued through ring opening and reduction of cyclopropane, followed by hydrogenation of the alkene (Scheme 13). High-pressure and colloidal Pt favoured the formation of thujopsane. The density of hydrogenated cedarwood oil was 0.917 g mL^−1^, which was higher than JP-8 and close to JP-10 (0.935 g mL^−1^). The presence of tricyclic sesquiterpenes was responsible for its high density. Similarly, the NHOC of hydrogenated cedarwood oil was 12% higher than JP-8 but 0.8% lower than JP-10.^91^

**Table 11:** Catalytic conversion of plant oils into SAF^a^

Oil	Reactor	Catalyst	Reaction conditions	Products	*ρ*	QL	QM	*η*	Ref.
Cedarwood oil	Batch	PtO_2_	459 g oil, 200 mL glacial acetic acid, 2.54 g catalyst, 120 °C, 400 psi H_2_, and 18 h	Thujopsane	0.917	39.129	—	54	91
Orange oil	Batch	Pt/Al_2_O_3_	1 mol oil, 100–200 mL ethanol, 4 g catalyst, 60 °C, 5.5 h, and 6 bar H_2_	1-*p*-menthene, *p*-menthane, 2-ethyl-6-methyl-1,5 heptadiene, *cis*-2,6-dimethyl-2,6-octadiene, and 2,6-dimethyl-2-octene	0.825	35	42.4	3	121
Turpentine	Flow	MMT K-10	5 mL turpentine, 250 mg catalyst, 5 mL heptane, 100 °C, and 24 h	Monomers (23%), dimers (66%), and trimers (11%)	0.93	39.29	—	—	114
	Flow	Nafion	10 mL turpentine, 200 mg catalyst, 5 mL heptane, 100 °C, and 24 h	Monomers (29%), dimers (70%), and trimers (1%)					114
	Flow	Nafion SAC-13	10 mL turpentine, 150 mg catalyst, 5 mL heptane, 100 °C, and 24 h	Monomers (12%), dimers (81%) and trimers (7%)					114
	Batch	Pt/Al_2_O_3_	4 g catalyst, 0.1 g palmitic acid as cocatalyst, 100 mL ethanol, 60 °C, 6 bar H_2_, and 4.5 h	Pinane, α-pinene, 1-*p*-menthene, *p*-menthane, iso-longifolene, iso-camphene, and 2,6-dimethyl octane	0.86	36.7	42.7	6	123
	Batch	SnCl_4_.5H_2_O	10 g turpentine, 1g catalyst, 90 °C, and 1 h	Limonene (∼10%), α-terpinene (5.5%), terpinolene (4.5%), γ-terpinene (2.5%), camphene (8.5%), menthane (3%), *p*-cymene (3%), bornyl chloride (6%), borneol (1.8%), fenchyl chloride (1%), dimers (40%), and trimers (6%)	—	—	—	—	126
	Batch	Mn_0.5_ [MIMPS]_2_ PW_12_O_40_	Dimerization: 8.6 g turpentine, 0.6 g catalyst, 10 mL toluene, 80 °C, 16 h. Hydrogenation: 3 wt% Pd/C, 3 Mpa H_2_, 160 °C, and 3 h	Dimers, camphene, limonene, α-terpinene, terpinolene, γ-terpinene, camphene, menthane, *p*-cymene, tricyclene, menthene, 3-carene, β-pinene, and others	0.91	38.9	—	26.5^b^	112
Oleoresin + turpentine	Batch	Ni/c-MWCNT	1 : 2 mass ratio of oleoresin : turpentine, 145 °C, 30 bar H_2_, and 1 h	Pinane (53.80%), longifolane (2.22%), caryophyllane (1.74%), dihydroabietic acid (19.67%), tetrahydroabietic acid (5.31%)	0.911^c^	—	—	—	125

a *ρ* = density at 15 °C, g mL^−1^, *η* = viscosity at −20 °C, mm^2^ s^−1^, QL = net heat of combustion per litre, MJ L^−1^, and QM = net heat of combustion per kg, MJ kg^−1^.

b At −10 °C.

c At 20 °C.

**Scheme 13:**
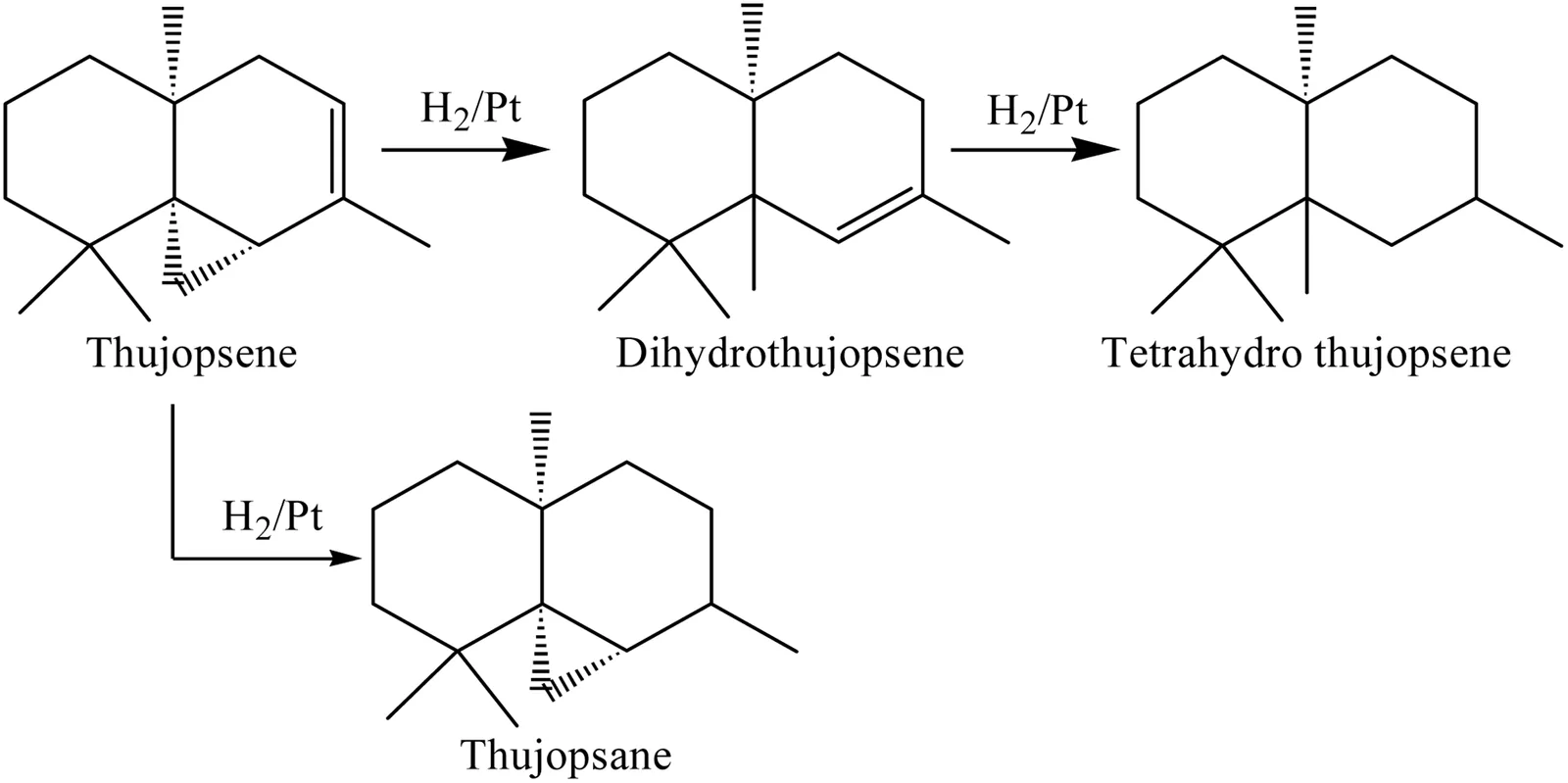
Hydrogenation of thjopsene.

### 5.2. Orange peel oil

Orange is the most cultivated fruit globally, accounting for approximately 60% of total citrus generation with an annual production of 100 million tons.^121^ The orange juice industry alone generates around 30 million tons of waste each year, most of which ends up in landfills, causing significant environmental problems and economic losses. The raw orange peel oil comprises 44.6% d-limonene, which can be enriched to 94% by steam distillation. Small amounts of other terpenoids, long-chain hydrocarbons, and oxygenated compounds are also present. Hydrogenation of orange peel oil and pure limonene over 1% Pt/Al_2_O_3_ showed 1-*p*-menthene as a major product, with only traces of *p*-menthane (Table 11).^121^ The hydrogenated distilled orange peel oil and neat d-limonene exhibited comparable density and crystallization points, satisfying the ASTM standard. However, hydrogenated orange peel oil showed higher viscosity, lower flashpoint, and lower heating value than hydrogenated limonene. On the other hand, it showed improved lubricity and a lower sooting tendency, with the latter decreasing linearly with conversion in both cases. Notably, purifying d-limonene from orange peel oil showed no improvement in fuel properties.^121^ Hydrogenated orange peel oil is a potential blending component with Jet A1 up to 15 vol%, in compliance with ASTM standard.^121^

### 5.3. Turpentine

Turpentine is a natural product obtained from various processes, primarily by distillation of resin harvested from living trees, mainly pines.^67^ It consists of monoterpenes and small quantities of sesquiterpenes. The major compounds in turpentine include α-pinene, β-pinene, and traces of d-limonene, camphene, and myrcene.^122^ Vacuum distillation of pine resin produces about 20% turpentine, while the bottom residue, known as rosin, accounts for 80% of the resin. Turpentine was traditionally used as a solvent in paints, but it was eventually replaced by petroleum-derived, cheaper alternatives. Consequently, turpentine has limited applications, and it is often considered a byproduct of industrial processes. Currently, the cheapest source of terpenes is crude sulphate turpentine, a byproduct of the pulp and paper industry.^67^ However, pure turpentine is unsuitable as SAF due to its high sooting tendency, low flash point, and poor oxidation stability. Hydrogenation of turpentine with Raney Ni and 1% Pt/Al_2_O_3_ in ethanol was thus carried out to saturate double bonds (Table 11).^123,124^ Hydrogenated turpentine met fuel specifications at 59% conversion, allowing its blending with Jet A1 up to 50 vol%. The cold-flow properties and lubricity were also achieved with a turpentine blend without additional additives.^123^

Catalytic hydrogenation of oleoresin-based turpentine to high-energy-density fuel was investigated over Ni-supported carboxylic multi-walled carbon nanotube (Ni/c-MWCNT) (Scheme 14).^125^ The initial oleoresin and turpentine mixture mainly consists of the monoterpene (pinene), sesquiterpene (caryophyllene and longifolene), and abietic acids. The pinene was hydrogenated to the pinane, while the abietic acid was hydrogenated mainly to dihydroabietic acid, with a small quantity of tetrahydroabietic acid. The freezing point, flash point, and density of hydrogenated oleoresin-based turpentine were aligned with the ASTM standards.^125^ Further, hydrogenated oleoresin-based turpentine exhibited higher calorific value, smoke point, and oxidative stability compared to the unsaturated mixture.

**Scheme 14:**
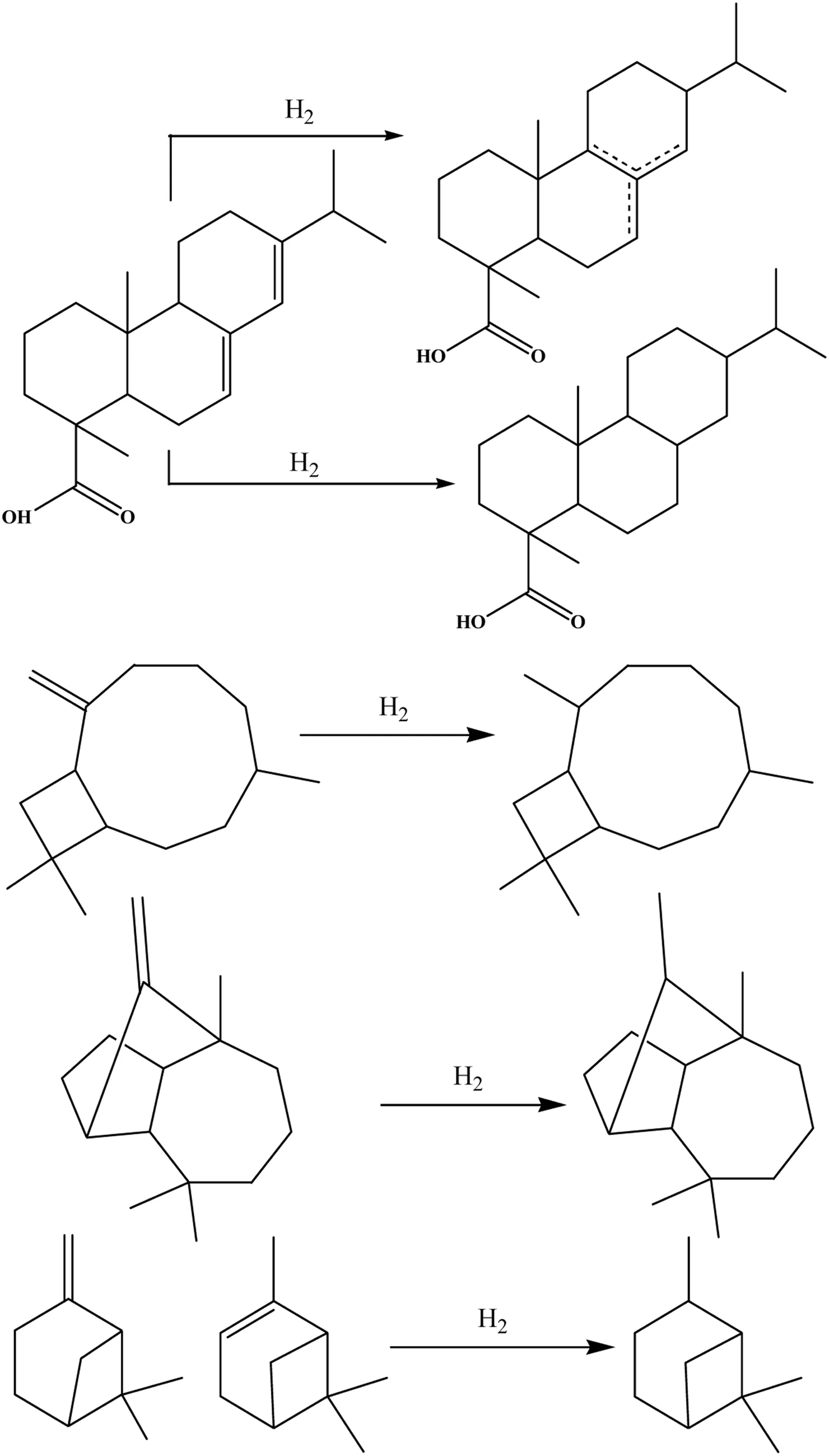
Hydrogenation of oleoresin–turpentine mixture (C_10_, C_15_, and C_20_ compounds).

Catalytic conversion of α-pinene, a major constituent of turpentine, into SAF was reported using stannic chloride molten-salt hydrate (SnCl_4_·5H_2_O) catalyst.^126^ The reaction was promoted by Brønsted acidity of the coordinated water ligand, which facilitated α-pinene isomerization and dimerization under solvent-free, mild conditions. At temperatures exceeding 90 °C, α-pinene underwent nearly complete conversion within one hour, yielding up to 61 wt% dimers, with enhanced fuel density. Isomer and dimer formation involved protonation, ring opening, and carbocation rearrangements. Hetero atoms (oxygen and chlorine) containing byproducts were also formed, arising from catalyst decomposition, specifically the dissociation of coordinated water or chloride ligands, leading to dimeric alcohols, ethers, and bornyl chloride. Strategies to mitigate side reactions include substituting coordinated water ligands with bidentate ligands like guaiacol or syringol to stabilize the stannic centre.^126^

## 6. Techno-economic and life cycle analysis

TEA and life cycle analysis (LCA) are essential for assessing economic and environmental sustainability. Recently, TEA was reported for manufacturing cyclo-paraffinic SAF components from 2000 metric tons of sorghum (dry) per day *via* integrated fermentative and catalytic approaches.^127^ Biomass was first pretreated with ionic liquids, with about 60% dissolution of lignin. Cellulose and hemicellulose were subsequently converted into fermentable sugars (glucose and xylose) by enzymatic hydrolysis, which were then metabolized by *E. coli* to produce five SAF precursors: limonene, 1,8-cineole, linalool, bisabolene, and *epi*-isozizaene (Scheme 15). Limonene, bisabolene, and *epi*-isozizene were hydrogenated to their respective paraffins, while 1,8-cineole and linalool were upgraded to limonane and RJ-4, respectively, *via* ring-opening/cyclization, dehydration, hydrogenation, dimerization, and isomerization reactions. The minimum selling price (MSP) per litre under the baseline scenario was $10.6, $6.4, $14.4, $6.2, and $8.9 for limonane from limonene, limonane *via* 1,8-cineole, RJ-4, bisabolane, and *epi*-isozizaane, respectively. Nevertheless, their prices were far from the targeted MSP of $0.66 per litre for Jet A.

**Scheme 15:**
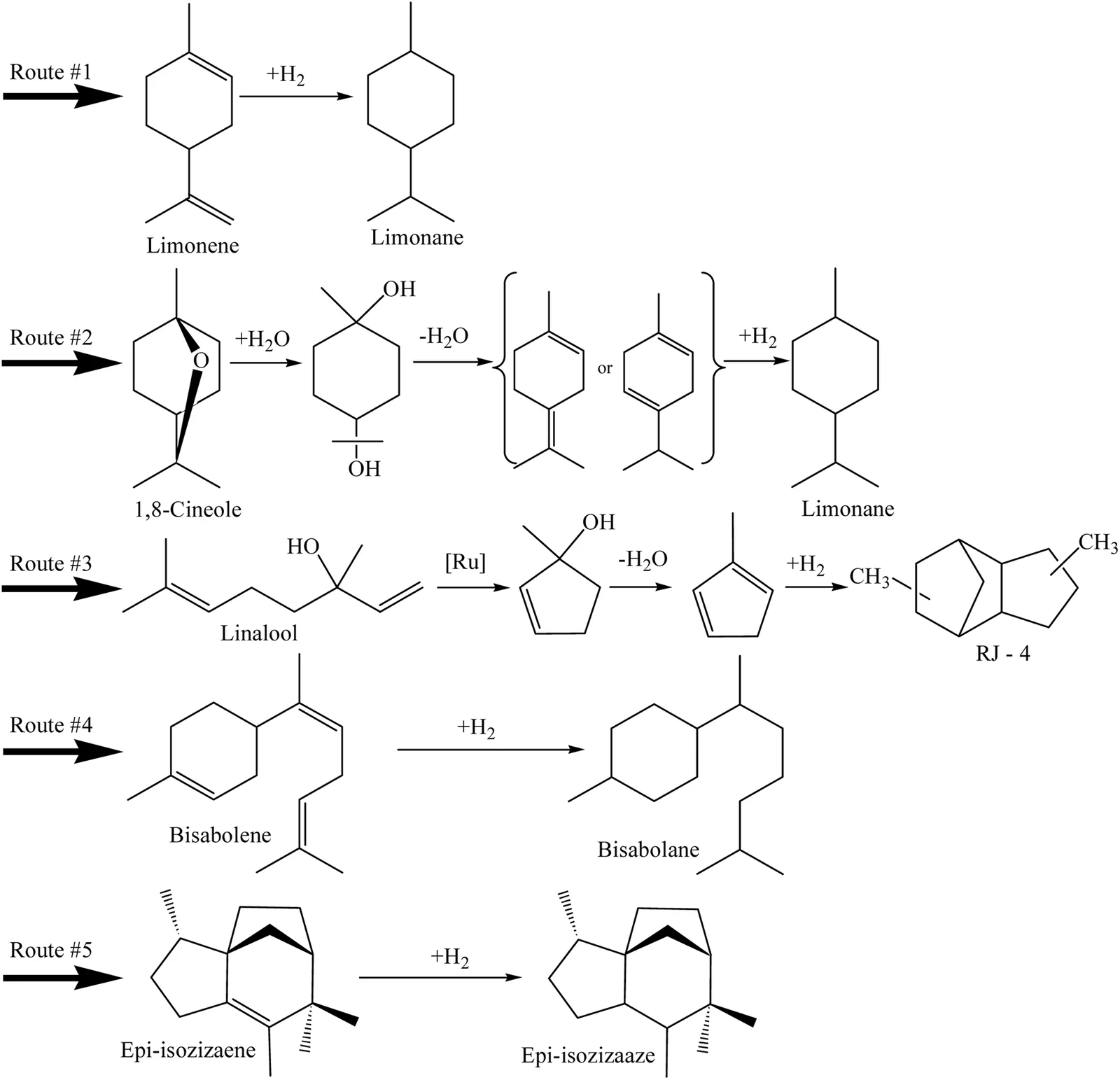
Catalytic conversion of isoprenoids into cyclo-paraffinic SAF components.

The SAF yield and MSP are significantly influenced by the feedstock and production technology.^24^ Recently, the MSP of SAF produced from jatropha oil *via* the HEFA route was $5.42 per gal ($1.43 per L) in the fruit scenario and $5.74 per gal ($1.52 per L) in the oil scenario.^128^ The disparity in the MSP was due to feedstock, capital, and energy costs, as well as co-product credit. On the other hand, MSP of SAF produced by FT synthesis of wood was reported as €1.24 per L ($1.45 per L), while it was €1.28–€1.49 per L ($ 1.49–$1.74 per L) from mixed alcohols *via* ATJ route under different scenarios.^129^ The MSP of SAF produced by hydroprocessing of microalgae was $8.44 per L.^130^ However, the MSP per litre of the terpene and terpenoid precursors could be reduced to $0.77, $0.91, $1.33, $0.82, and $0.73 by process optimization/intensification with near-theoretical yields.^127^ The higher MSP of RJ-4 was due to the complex manufacturing process. GHG mitigation costs for these SAFs were estimated at $29 per metric ton of CO_2_, which was nearly double the current cap-and-trade market price in California.^127^

## 7. Challenges and future directions

Terpenes and terpenoids are a diverse class of natural hydrocarbons sourced from plants, essential oils, and resins, or can be produced biologically. They are promising feedstock for producing SAF enriched with high-density branched and cyclic paraffins.^131–133^ However, several challenges remain in this approach, as discussed below.

• Availability, high cost, and process scalability are major challenges for these feedstocks.^36,67,134^ In 2019, global turpentine production from biomass and various industries was 316 000 tons, with approximately 185 000 tons coming from the paper industry alone.^67,123^ Additionally, 125 000 tons were obtained from pine resin, and 6000 tons were derived from stump wood. *Eucalyptus* spp. is a broad-leaved tree and cultivated on nearly 22 million hectares around the globe, but its mass production conflicts with food security.^135^ Recently, short-rotation *eucalyptus* spp. leaves showed economic viability for producing SAF.^136^ For an annual coppice system of eucalyptus cultivation for maximum leaf production with the lowest simulated price of $110 per dry ton, it can annually produce 204, 51, and 75 million litre of fuel, JP-10, and Jet A within 10 years, respectively. However, fluctuations in feedstock quality may adversely impact SAF production, while inconsistent supply can lead to downtime.^136^

• Feedstock logistics, emerging technologies, economic insecurity, and inconsistent regulation are additional challenges. These hurdles can be overcome by adopting mixed feedstock, decentralized biorefinery, accurate model-based prediction, optimization, and supportive policies.^137^

• The production of SAF-range alkanes from terpenes, terpenoids, and essential oils involves multiple reactions, necessitating the development of stable and selective catalysts.^73,75^ An understanding of the role of each product on fuel properties is also crucial in developing SAF technology. Additionally, the production of broader classes of terpenes, their performance evaluation, and the correlation of their structures with fuel properties should be thoroughly studied.^138^

• The conversion of terpenoids, terpenes, and plant oils to SAF involves hydrogen-consuming hydrogenation and HDO reactions, which can affect production costs and the process sustainability. Therefore, optimal reaction parameters and efficient catalysts are essential for reducing the hydrogen consumption. Additionally, the use of green hydrogen in SAF production should be emphasized to minimize lifecycle carbon emissions.

• Quantum mechanical calculations and machine learning can help design high-performance cycloparaffins based on strain energy and electronic structure. However, the limited availability of reliable property data of SAF components restricts modelling accuracy. Some terpenes or terpene-derived hydrocarbons have higher densities than ASTM specifications.^115^ The cold flow properties also strongly depend on the structure and nature of the precursor.

## 8. Summary

The catalytic conversion of terpenes, terpenoids, and plant oils produces a diverse range of cycloparaffins suitable for SAF applications. Fig. 5 summarizes the density, viscosity, and specific NHOC of various cycloparaffins and blends discussed in this review and benchmarked against the Jet A/A1 and ASTM-approved SAFs. These results demonstrate that most of the terpene-derived SAF blends are within or near the Jet A/A1 density range. On the other hand, the density of terpene dimers is well above the Jet A/A1 density range. Their density is too high for standalone use as an aviation fuel. However, they are well-suited to high-density military/specialty applications, such as RJ-4-type ramjet or missile fuels.

**Fig. 5:**
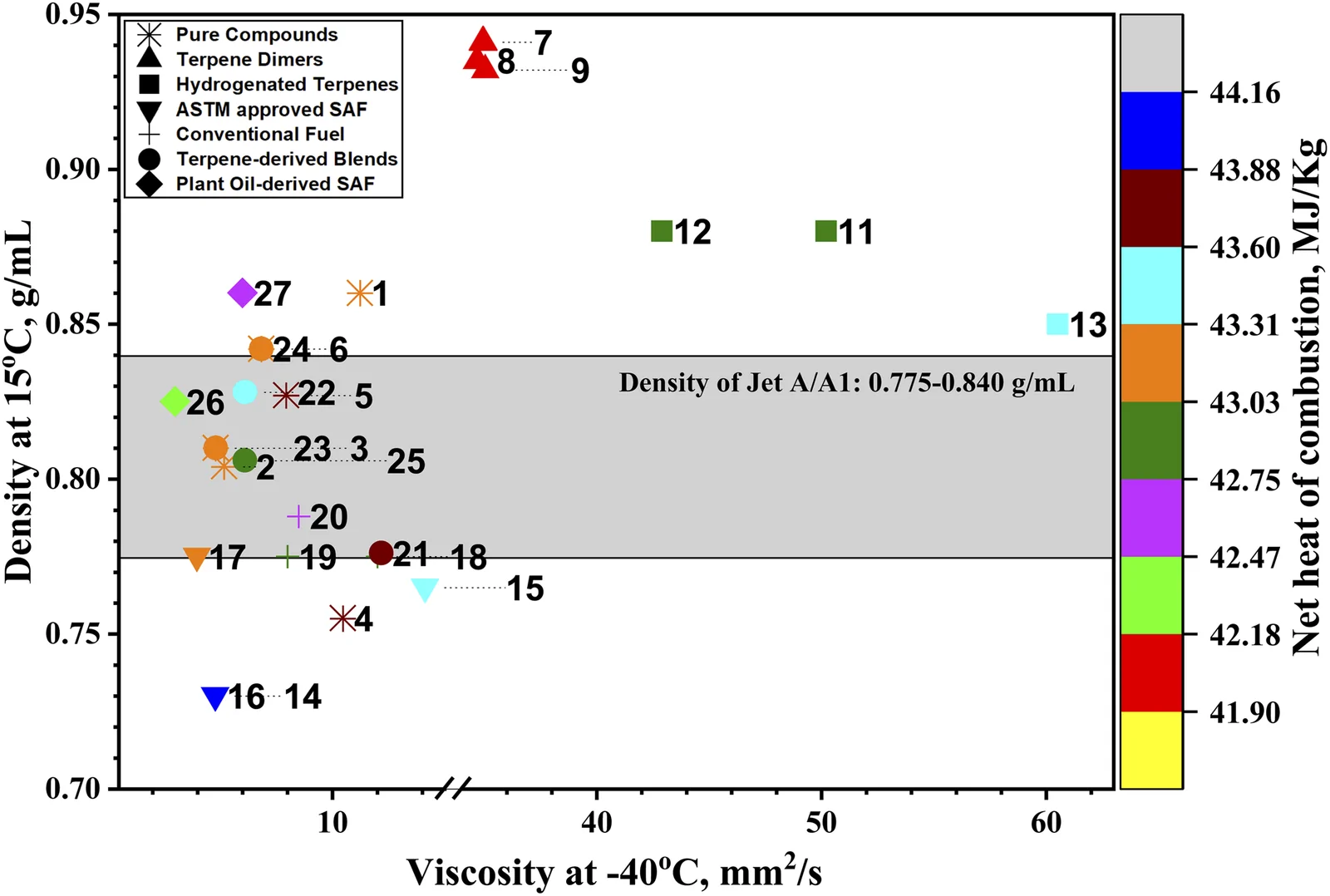
Density, viscosity, and NHOC for various terpene and terpenoid-derived SAF components. Viscosity is reported at 40 °C for 7–10 and at −20 °C for 11–13, 19–20, ASTM-approved SAFs, and plant oil-derived SAF. (1) α-Pinane, (2) *p*-menthane, (3) sabinane, (4) 5-methylundecane, (5) 1,4-dimethylcyclooctane, (6) carane diastereomers, (7) camphene dimers, (8) α-pinene dimers, (9) β-pinene dimers, (10) limonene dimers, (11) hydrogenated valencene, (12) hydrogenated premnespirodiene, (13) hydrogenated β-caryophyellene, (14) HEFA-SPK, (15) HFS-SIP, (16) ATJ-SPK, (17) CH, (18) Jet A/A1, (19) JP-8, (20) JP-5, (21) ∼20% pinane + 80% 5-MU, (22): 78% thujane + 21% pinane, (23) 76% tetrahydroabinene + 23% pinane, (24): 97% carane + 3% 1,1,4-trimethylcycloheptane, (25): 90% 1,1,4-trimethylcycloheptane + 4% carane + 6% *p*-menthane; (26) orange oil, and (27) turpentine.

## 9. Conclusions

Terpenoids, terpenes, and plant oils are biologically derived or natural resources with an in-built cycloparaffin structure. These feedstocks can provide cycloparaffin-rich high-density SAF with improved volumetric heat of combustion, low freezing point, and reduced viscosity. The processing of terpenoids to SAF-range alkanes involves the removal of oxygen by acid-catalysed dehydration or deoxygenation, followed by oligomerization and hydrogenation. On the contrary, oxygen-free terpenes are processed *via* hydrogenation, dimerization, or their combination. However, the carbon chain length of cyclo-paraffins obtained from terpenes and terpenoids is either lower (C_10_) or higher (C_20_ for dimerization) than the desirable number for SAF (C_8_–C_16_). Several products derived *via* HDO of terpenoids combined with hydrogenation of monoterpenes largely meets Jet A specifications, including density, viscosity, and NHOC. The dimerization is an indispensable strategy to obtain high-density SAF for military and speciality applications. Blending different components or cyclopropanation of terpenes are alternative approaches to match the molecular weight and fuel properties. Further studies are also needed to develop scalable cyclopropanation technology using heterogeneous catalysts. Producing SAF from plant oils is another approach for deriving mixed hydrocarbons with improved properties. However, this approach is limited due to the scarcity and high cost of feedstock. The current state-of-the-art results are not economically favourable for the biological production of terpenoids and terpenes from cellulosic biomass and their downstream conversion to SAF due to low titters. Future research should focus on improving titter, yield, and productivity through metabolic engineering. The studies should also focus on producing cycloparaffin-rich SAF from plant oils and evaluate their economic and environmental sustainability using TEA and LCA tools.

## Author contributions

DA and BSS: writing – original draft, writing – review & editing; SG, AT and MS: conceptualization, writing – review & editing; AX: writing – review & editing; VK: conceptualization, analysis, writing – review & editing; SKM: conceptualization, analysis, methodology, supervision, writing – review & editing, project administration.

## Conflicts of interest

There are no conflicts to declare.

## Abbreviations

ATJ: Alcohol-to-jet
CH: Catalytic hydrothermolysis synthetic kerosene
FOG: Fats, oil, and grease
FT: Fischer–Tropsch
GHG: Greenhouse gas
HDO: Hydrodeoxygenation
HEFA: Hydro-processed esters and fatty acids
LCA: Life cycle analysis
MJ: Megajoule
MM: Million
MSP: Minimum selling price
NHOC: Net heat of combustion
SAF: Sustainable aviation fuel
SIP: Synthetic iso-paraffins
SPK: Synthetic paraffinic kerosene
SKA: Synthetic kerosene with aromatics
TEA: Techno-economic analysis
TFA: Trifluoracetic acid

## Supplementary Material

RA-016-D6RA06326H-s001

## Data Availability

No primary research results, software or code have been included and no new data were generated or analysed as part of this review. Supplementary information (SI): properties of SAFs. See DOI: https://doi.org/10.1039/d6ra06326h.
