# Supplementary material for: Cycloparaffin-rich high density sustainable aviation fuel: pathways from terpenes, terpenoids, and plant oils
Source: RSC Adv. 2026 Oct 1;16(46):58728–61. doi: 10.1039/d6ra06326h (PMC13629306; doi:10.1039/d6ra06326h)
Supplement: Supplementary file 1 [file RA-016-D6RA06326H-s001.pdf]

Supplementary Information (SI) for RSC Advances.
This journal is © The Royal Society of Chemistry

Supplementary information

Cycloparaffin-rich high density sustainable aviation fuel: Pathways from terpenes, terpenoids, and plant oils

Dandu Anitha [1,#], Bhushan S. Shrirame [1,#], Siddharth Gadkari [2], Atthasit Tawai [3], Malinee Sriariyanun [3], Ao Xia [4], Vinod Kumar [5, 6*], Sunil K. Maity [1,*]

[1] *Department of Chemical Engineering, Indian Institute of Technology Hyderabad, Kandi, Sangareddy-502284, Telangana, India*

[2] *School of Chemistry and Chemical Engineering, University of Surrey, Guildford GU2 7XH, UK*

[3] *The Sirindhorn International Thai-German Graduate School of Engineering, King Mongkut's University of Technology North Bangkok, Bangkok 10800, Thailand*

[4] *Institute of Engineering Thermophysics, School of Energy and Power Engineering, Chongqing University, Chongqing 400044, China*

[5] *Magan Centre for Applied Mycology, Faculty of Engineering and Applied Sciences, Cranfield University, Cranfield MK43 0AL, United Kingdom*

[6] *Department of Engineering Technology, Cullen College of Engineering, University of Houston, Sugarland, TX 77479, USA*

[#] *Equal first author contribution*

***Corresponding authors:**

1. *Dr. Vinod Kumar, Ph: +44 (0) 1234754786, E-mail: vinod.kumar@cranfield.ac.uk*
2. *Prof. Sunil K. Maity, Ph: +91-40-23016202, E-mail: sunil_maity@che.iith.ac.i*

Table S1

Properties of currently available SAF [1–4].

**Properties**	**FT-SPK**	**HEFA-SPK**	**HFS-SIP**	**FT-SPK/A**	**ATJ-SPK**	**CH**	**ATJ-SKA**
Molecular formula	$C_{11}H_{23.7}$	$C_{11.3}H_{24.4}$	$C_{15}H_{32}$	-	$C_{12.5}H_{27.1}$	$C_{11.2}H_{21.5}$	$C_{11.1}H_{23.5}$
n-Paraffins, vol%	99	9.1-12.8	0	1.9-9.4	0	44.0	85.0
iso-Paraffins, vol%	-	86.9 - 89.4	96.4	52.9-67.4	99.8	3.9	-
Olefins, vol%	-	0.1	0.2	1.3	0–2.3	0.8–5.1	1.5
Cycloparaffins, vol%	0.5	0.3–0.9	0–1.3	8.8–26.1	0.2	32.9–57.3	0
Aromatics, vol%	0–0.5	0-<0.3	0-0.1	1.2–21.9	0–0.7	2.6–24.2	11.7–18.1
Sulfur, wt%	0–0.002	0-0.0015	0.0002	0.0001	< 0.001	< 0.001–0.03	0.00003
Water, mg/kg	-	75	22–80	-	36	39	0
Acidity, mg KOH/g	0.001–0.004	0.001	0.002–0.005	0.005	0.002–0.007	0.001–0.014	0.003
Freezing point, °C	<-66 to -43	<-77 to -40	<-100 to –47	-72	<-82 to -40	<-57 to -39	<-80 to -47
Viscosity at –20 °C, mm^2/s	2.4–6.1	3.3–5.5	14.1	3.1–4.0	4.8–8.0	3.5–4.0	3.4–3.9
Net heat of combustion, MJ/Kg	43.8–46.7	43.2–44.3	43.5–44.5	43.5–43.6	43.2–44.1	42.9–43.6	43.2–43.6
Smoke point, mm	28-50	50-53	-	24	27-35	21->30	23-27
Density at 15 °C, kg/m^3	735-765	749-802	770-786	786–805	750-774	793-805	779-786
Flashpoint, °C	40-70	39-55	96-108	47-50	38-60	41-48	38–48.5
Initial boiling point, °C	144-179	131–164.5	237-247	162-164	174-179	150-152	160-165
Final boiling point, °C	195-275	243-274	258	242-243	250-300	248-268	250-260

References

1 *ASTM International*, Standard Specification for Aviation Turbine Fuel Containing Synthesized Hydrocarbons, 2024, DOI: 10.1520/D7566-24A.

2 F. Wang and D. Rijal, *Energy Fuels*, 2024, **38**, 4904–4920.

3 P. Kurzawska-Pietrowicz and R. Jasiński, *Energies*, 2024, **17**, 3890.

4 U. A. Makhova, M. A. Ershov, A. V. Ilin, V. D. Savelenko, N. O. Burov, M. M. Lobashova, E. A. Sharin, A. A. Shevtsov, A. O. Vikhritskaya, V. M. Kapustin and T. M. M. Abdellatief, *Process Saf. Environ. Prot.*, 2024, **188**, 905–916.
